# Efficient oxygen evolution electrocatalysis in acid by a perovskite with face-sharing IrO_6_ octahedral dimers

**DOI:** 10.1038/s41467-018-07678-w

**Published:** 2018-12-07

**Authors:** Lan Yang, Guangtao Yu, Xuan Ai, Wensheng Yan, Hengli Duan, Wei Chen, Xiaotian Li, Ting Wang, Chenghui Zhang, Xuri Huang, Jie-Sheng Chen, Xiaoxin Zou

**Affiliations:** 1 State Key Laboratory of Inorganic Synthesis and Preparative Chemistry, College of Chemistry, Jilin University, 130012 Changchun, People’s Republic of China; 2 College of Materials Science and Engineering, Jilin University, 130022 Changchun, People’s Republic of China; 3 Laboratory of Theoretical and Computational Chemistry, Institute of Theoretical Chemistry, Jilin University, 130023 Changchun, People’s Republic of China; 4 National Synchrotron Radiation Laboratory, University of Science and Technology of China, 230029 Hefei, Anhui People’s Republic of China; 5 School of Chemistry and Chemical Engineering, Shanghai Jiao Tong University, 200240 Shanghai, People’s Republic of China

## Abstract

The widespread use of proton exchange membrane water electrolysis requires the development of more efficient electrocatalysts containing reduced amounts of expensive iridium for the oxygen evolution reaction (OER). Here we present the identification of 6H-phase SrIrO_3_ perovskite (6H-SrIrO_3_) as a highly active electrocatalyst with good structural and catalytic stability for OER in acid. 6H-SrIrO_3_ contains 27.1 wt% less iridium than IrO_2_, but its iridium mass activity is about 7 times higher than IrO_2_, a benchmark electrocatalyst for the acidic OER. 6H-SrIrO_3_ is the most active catalytic material for OER among the iridium-based oxides reported recently, based on its highest iridium mass activity. Theoretical calculations indicate that the existence of face-sharing octahedral dimers is mainly responsible for the superior activity of 6H-SrIrO_3_ thanks to the weakened surface Ir-O binding that facilitates the potential-determining step involved in the OER (i.e., O* + H_2_O → HOO* + H^+^ + *e*_¯_).

## Abstract (web summary)

While splitting water may provide a renewable source of carbon-neutral energy, the water oxidation half-reaction is sluggish and the materials needed show poor stability. Here, authors demonstrate an unusual iridium-based oxide to perform high-efficiency oxygen evolution in acid with good stability.

## Introduction

The oxygen evolution reaction (OER) is the primary reaction that occurs at the anode in many electrochemical energy conversion processes, such as water splitting, CO_2_ reduction, N_2_ fixation, etc^1–3^. Due to its multi-proton/electron-coupled kinetics, the OER is a quite sluggish half-reaction, and often has a crucial role in the overall efficiency of these electrochemical processes^1,4,5^. Therefore, over the past several years, considerable effort has been devoted to the search and synthesis of efficient electrocatalysts that can significantly lower the kinetic barriers for OER. Whereas a large number of oxygen evolution electrocatalysts based on a wide range of transition metals work well under alkaline conditions^6–13^, only the few of the recently developed electrocatalysts are found to be effective for OER in acidic media^14–23^. Despite the difficulties, there are strong demands for the OER in acidic pH regime. For instance, proton exchange membrane (PEM) water electrolysis is an attractive and advanced route for sustainable hydrogen production^24^, especially when coupled with some renewable energy systems. PEM water electrolysis offers many advantages, such as high current densities, ultrahigh gas purities, low ohmic losses and good compactness^24^. But this technique requires corrosion-resistant electrocatalysts that can operate efficiently in strongly acidic media.

The reasons for the slow development of OER electrocatalysts operating in acid can be reflected by the facts that: first, the great majority of promising electrocatalysts, including those can work well in alkaline media, are chemically unstable in acid^6–13^; second, the corrosion/decomposition of electrocatalysts in acid always get worse under a strongly oxidative electrocatalysis condition; third, even for the few with relatively good catalytic stability in acid, uncontrolled surface reconstruction often take place during electrocatalysis, so that the accurate elucidation of the atomic basis for catalytic properties is very difficult^16,17^. At present, iridium-based oxides, especially IrO_2_, are generally considered the only electrocatalysts with reasonable activity and stability for the acidic OER. So, considering the high cost and low Earth abundance of iridium, researchers are in pursuit of corrosion-resistant, structurally stable, more active oxygen evolution electrocatalysts containing reduced iridium amount.

Herein we identify 6H-SrIrO_3_, containing 27.1 wt% less iridium than IrO_2_, as a highly efficient electrocatalyst with excellent structural stability for OER in acid. The material gives the lowest overpotential at 10 mA cm_geo_^−2^ (current densities per geometric area), and exhibits the highest iridium mass activity for the acidic OER among the iridium-based oxide catalysts reported recently. To the best of our knowledge, an iridium-based compound (or an unusual perovskite oxide) with face-sharing IrO_6_ octahedral subunits has never been reported to effectively electrocatalyze OER in acid. In addition, 6H-SrIrO_3_ has never been studied as a catalyst in any chemical reactions.

## Results

### Crystal structure and electrical conductivity of 6H-SrIrO_3_

**Fig. 1:**
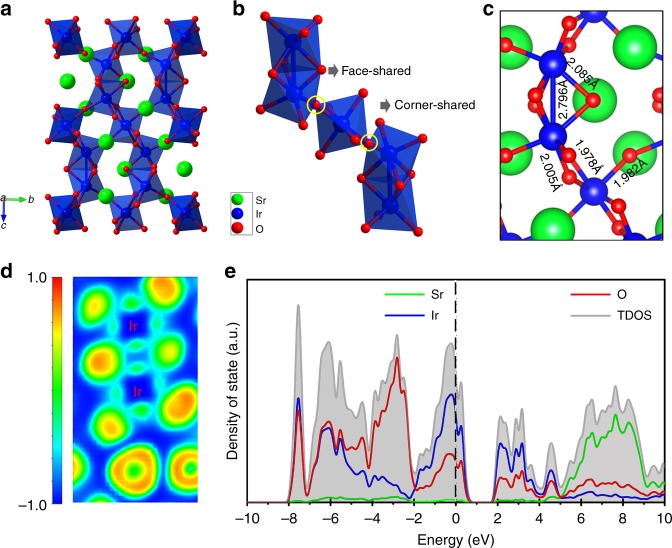
Crystal structure and electronic structure of 6H-SrIrO_3_. **a** Crystal structure of 6H-SrIrO_3_. **b** A local connection pattern of IrO_6_ octahedra, in which face-sharing IrO_6_ octahedral dimers and corner-sharing, isolated IrO_6_ octahedron are shown. **c** Local ball-and-stick model of 6H-SrIrO_3_, in which typical Ir–Ir and Ir–O bond lengths are presented. **d** Plot of electron location function for Ir–Ir bonding in 6H-SrIrO_3_. **e** Density of states of 6H-SrIrO_3_, in which the Fermi level is 0 eV

6H-SrIrO_3_ is the thermodynamically stable polymorph of SrIrO_3_, and adopts a monoclinic distortion structure of the hexagonal form of perovskite BaTiO_3_ (Fig. 1a)^25^. It consists of alternating face-sharing IrO_6_ octahedral dimers and corner-sharing, isolated IrO_6_ octahedra along the *c* axis (Fig. 1b). Correspondingly, there are two types of Ir atoms in 6H-SrIrO_3_. One is in the face-sharing IrO_6_ octahedral dimers, and the other is in the corner-sharing, isolated IrO_6_ octahedra (Fig. 1c). It is remarkable that there is Ir–Ir metallic bonding in face-sharing IrO_6_ octahedra, as revealed by the very short Ir(I)–Ir(I) distance (2.796 Å). This can be further supported by the electron location function (ELF)^26,27^. As presented in Fig. 1d, the correlative ELF values with a range of 0.4 to 0.5 distribute between the two neighboring Ir atoms in face-sharing IrO_6_ dimers, indicating that Ir–Ir bond is metallic in character. (See details about ELF in Methods). Besides Ir–Ir metallic bonding, there are obviously weaker Ir–O bonds in the face-sharing IrO_6_ octahedral dimers in comparison with those in the corner-sharing, isolated IrO_6_ octahedra. As shown in Fig. 1c and Supplementary Figure 1, the Ir–O bond lengths in the former, ranging from 2.005 to 2.085 Å, are much larger than those in the latter (1.978–1.982 Å, close to those in IrO_2_). The face-sharing IrO_6_ octahedra and the unique bonding features in them, including the Ir–Ir metallic bonding and the weak Ir–O bonding, mark 6H-SrIrO_3_ off from other Ir-based oxide catalysts reported previously (e.g., IrO_2_, see its crystal structure in Supplementary Figure 2)^15–22^.

The computed density of state (DOS) for 6H-SrIrO_3_ reveals that this material has intrinsic metallic conductivity^25,28^, which is reflected by the density of state from Ir and O atoms crossing the Fermi level (Fig. 1e). This is further supported by the experimental result (Supplementary Figure 3). The electrical resistivity of 6H-SrIrO_3_ was measured to be ~2.7 × 10^−4^ Ω m at room temperature. The metallic conductivity of 6H-SrIrO_3_ is believed to be beneficial for electrochemical applications.

### Synthesis and electrochemical properties for OER in acid of 6H-SrIrO_3_

**Fig. 2:**
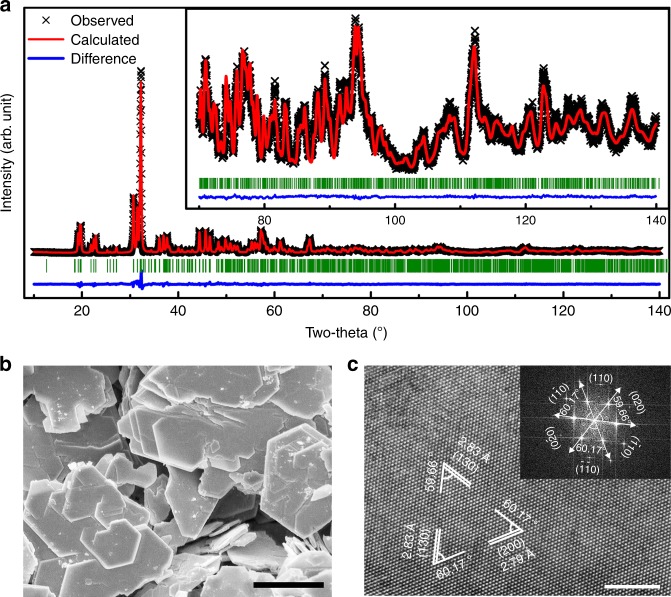
Structural characterizations of 6H-SrIrO_3_. **a**, XRD pattern with a refinement plot of 6H-SrIrO_3_. **b**, SEM image of 6H-SrIrO_3_. Scale bar, 2 µm. **c**, HRTEM image of 6H-SrIrO_3_. Scale bar, 5 nm. Inset: the corresponding fast Fourier transform image

Encouraged by the unique crystal structure of 6H-SrIrO_3_, we synthesized this material and attempted to explore its potential electrocatalytic activity for OER in acid. The observed powder X-ray diffraction (XRD) pattern is almost in agreement with the fitting plot (Fig. 2a), revealing that the material is composed of high purity 6H-SrIrO_3_. The refinement results are listed in Supplementary Table 1. Scanning electron microscopy (SEM) images (Fig. 2b and Supplementary Figure 4) show that the material has micron-sized, plate-like particles with a dominant thickness distribution of 30–60 nm. Additionally, chemical mapping and energy dispersive X-ray spectroscopy (EDS) show that the Sr and Ir elements are homogeneously distributed over the material, with a Sr:Ir atomic ratio of ca. 1:1 (Supplementary Figure 5). X-ray photoelectron spectroscopy (XPS) result further reveals a Sr:Ir atomic ratio of ca. 1:1 for 6H-SrIrO_3_. In the high resolution transmission electron microscopy (HRTEM) images (Fig. 2c and Supplementary Figure 6), three sets of lattice fringes are shown, giving interplanar distances of 0.283, 0.279, and 0.283 nm corresponding to the (130), (200), and ($\overline 1$30) crystallographic planes of 6H-SrIrO_3_, respectively. The observed angles between these crystallographic planes match well with the theoretical values. These results further suggest that the exposed facet of micron-sized 6H-SrIrO_3_ plates is {001}. The exposure of this facet is also confirmed by the fast Fourier transform image (Fig. 2c, inset). Furthermore, high-angle annular dark field (HAADF) STEM image (Supplementary Figure 7) of the edge of a 6H-SrIrO_3_ particle again reveals that the 6H-SrIrO_3_ is highly crystalline.

Before the electrocatalytic studies, we first tested the chemical stability of 6H-SrIrO_3_ in strongly acidic media (0.5 M H_2_SO_4_). XRD and TEM results reveal that 6H-SrIrO_3_ retains its crystal structure well, even after its exposure in 0.5 M H_2_SO_4_ over 48 days (Supplementary Figure 8). In addition, after such a long time testing, there is no detectable leached Ir species and only ~1.3% of total Sr content leached in the acidic solution. These results suggest that 6H-SrIrO_3_ has excellent chemical stability in acid.

**Fig. 3:**
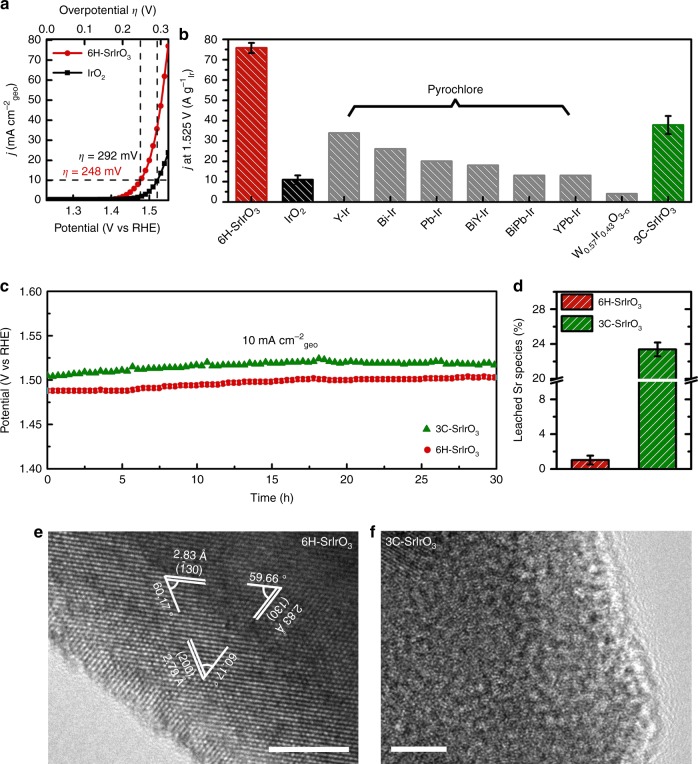
Electrocatalytic properties for OER of 6H-SrIrO_3_. **a** Polarization curves of 6H-SrIrO_3_ and IrO_2_ in 0.5 M H_2_SO_4_ solution with 85% iR-compensations. The current densities are normalized by the geometric area. **b** Comparison of mass activity, normalized by the mass of iridium at 1.525 V vs. RHE, of 6H-SrIrO_3_, IrO_2_, 3C-SrIrO_3_, and some Ir-containing catalysts reported recently. The error bar represents standard deviation based on five measurements. **c** Chronopotentiometric curves for OER in the presence of 6H-SrIrO_3_ and 3C-SrIrO_3_ in 0.5 M H_2_SO_4_ solution at 10 mA cm^−2^_geo_ (without iR compensations). **d** Percentage of total Sr content leached (or deviated from the stoichiometry) in the solution after 30 h-long catalytic stability test in the presence of 6H-SrIrO_3_ and 3C-SrIrO_3_. **e**, **f** HRTEM images for 6H-SrIrO_3_ and 3C-SrIrO_3_ after 30 h-long catalytic stability test. Scale bars, 5 nm

Next, we evaluated the electrocatalytic properties of 6H-SrIrO_3_ toward OER in acidic media (0.5 M H_2_SO_4_). For comparative purpose, we also synthesized IrO_2_ nanoparticles with a particle size of 10–20 nm (see structural characterizations in Supplementary Figure 9) and studied their catalytic activity for OER. As shown in Fig. 3a, both 6H-SrIrO_3_ and IrO_2_ exhibit remarkable electrocatalytic activity toward OER, and the former is more active than the latter. For example, at 1.525 V, the electrocatalytic activity of 6H-SrIrO_3_ (measured by the current densities per the geometric area) is almost four times higher than that of IrO_2_. Additionally, 6H-SrIrO_3_ produces a current density of 10 mA cm^−2^_geo_ at an overpotential (*η*) of 248 mV, whereas IrO_2_ gives the same current density at a higher overpotential (300 mV). The results also reveal that 6H-SrIrO_3_ is among the most active Ir-based oxide electrocatalysts for OER in acid (see Supplementary Table 2), although there might be the differences in protocols, such as catalyst loading, use of binder and electrolyte, employed in their electrocatalytic tests. For example, some Pb-, Bi-, or Y-containing, Ir-based pyroclore compounds usually give a current density of 10 mA cm^−2^_geo_ at overpotantials higher than 340 mV^19–22^, and Ir–W bimetallic oxide catalysts with an optimal composition require an overpotential of 370 mV for the acidic OER^22^. Moreover, a perovskite-type La_2_LiIrO_6_ catalyst can produce a current density of 10 mA cm^−2^_geo_ at an overpotential of ca. 300 mV^17^, but this material suffers from the serious surface reconstruction, resulting in the generation of IrO_2_ nanoparticles, during electrocatalysis.

Besides exhibiting good catalytic activity per geometric area, 6H-SrIrO_3_ also shows good mass activity (normalized by the mass of iridium) for the acidic OER. As shown in Fig. 3b, 6H-SrIrO_3_ gives the highest mass activity for OER in acid among the iridium-based oxide electrocatalysts reported recently^15,16,18–22^. The mass activity of 6H-SrIrO_3_ is about seven times as high as that of IrO_2_, and is 2.2–5.8 times higher than those of Ir-based pyroclore compounds. The mass activity of 6H-SrIrO_3_ is even around twenty times higher than the Ir–W bimetallic oxide catalyst. Moreover, it is worth noting that the superior catalytic activity of 6H-SrIrO_3_ does not correlate with its surface area. The surface area (obtained with N_2_ adsorption method) of 6H-SrIrO_3_ is typically lower by more than an order of magnitude in comparison with the Ir-based catalysts mentioned above (see Supplementary Table 2). For example, the IrO_2_ sample has a larger BET surface area (19.8 m^2^ g^−1^) than 6H-SrIrO_3_, due to the former’s smaller particle size (see Supplementary Figures 4 and 9). This is in agreement with the effective electrochemical active surface area of IrO_2_, which is measured to be about three times larger than that of 6H-SrIrO_3_ (Supplementary Figure 10).

### Comparison of electrocatalytic properties for OER between 6H-SrIrO_3_ and 3C-SrIrO_3_

In order to further assess the catalytic properties of 6H-SrIrO_3_, we synthesized 3C-phase SrIrO_3_ perovskite (3C-SrIrO_3_) as a reference material for comparative studies. 3C-SrIrO_3_ adopts a well-known pseudo-cubic structure, in which all the IrO_6_ octahedra are corner-shared (see its crystal structure in Supplementary Figure 11)^29^. Additionally, the thinfilms of 3C-SrIrO_3_ were recently demonstrated to give the best intrinsic catalytic activity for OER in acid^16^, due to the in situ generation of amorphous IrO_x_ on the surface by serious strontium leaching during electrocatalysis.

The experimental procedures are almost the same for the synthesis of 6H-SrIrO_3_ and 3C-SrIrO_3_ (see experimental details in Methods). In order to explore the reasons why the addition of more critic acid in the synthesis can favor the formation of metastable 3C-SrIrO_3_ over 6H-SrIrO_3_, we first compared the Ir4*f* XPS spectra of IrO_2_, 6H-SrIrO_3_ and 3C-SrIrO_3_ (Supplementary Figure 12a). The results reveal that the Ir 4*f*_5/2_ and Ir 4*f*_7/2_ XPS peaks for 3C-SrIrO_3_ appear at the higher binding energies compared with IrO_2_ and 6H-SrIrO_3_. This demonstrates that the average oxidation state of iridium for 3C-SrIrO_3_ is higher than 4+^30^. The higher average oxidation state of iridium are believed to be favorable for the formation of 3C-SrIrO_3_. This argument is mainly based on recent studies, in which low-valent heteroatom doping (e.g., Co, Mg, Zn, etc.) can lead to the increase in the oxidation state of iridium and the stabilization of 3C-SrIrO_3_^28,31,32^. In view of the difference in synthetic systems between our work and the previous studies^28,31,32^, we attempted to synthesize 3C-phase SrIrO_3_ by low-valent heteroatom doping (cobalt as dopants used in this work) with the experimental procedures that were used for the synthesis of 6H-SrIrO_3_ (see experimental details in Methods). The results show that in this case, the formation of the 3C-phase is indeed preferable, and the resulting material also has a higher oxidation state of iridium (Supplementary Figure 12b). Next, we compared the thermogravimetric (TG) and differential thermal analysis (DTA) in air of the precursors of 6H-SrIrO_3_ and 3C-SrIrO_3_. As shown in Supplementary Figure 13a, the organic components in the precursors are oxidatively decomposed in air at elevated temperature (ca. 500 °C), and this process is highly exothermic. The presence of more critic acid (i.e., the precursor for the synthesis of 3C-SrIrO_3_) is also found to result in more heat released at elevated temperature. The more released heat in the synthesis should favor the generation of high-valent iridium species. This is confirmed by the XPS results (Supplementary Figure 13b), which show that the sample obtained by calcining the precursor of 6H-SrIrO_3_ at 500 °C contains zero-valent and four-valent Ir species, whereas the sample obtained by calcining the precursor of 3C-SrIrO_3_ at 500 °C contains four-valent and five-valent Ir species. Overall, the presence of more critic acid favors the formation of 3C-SrIrO_3_ by facilitating the generation of the more oxidized iridium species.

The 3C-SrIrO_3_ material we synthesize contains of micron-sized particles with a small surface area comparable with that of 6H-SrIrO_3_ (Supplementary Figures 14, 15 and Table 2). The 3C-SrIrO_3_ material has a Sr:Ir atomic ratio of 0.82:1 and a certain amount of IrO_x_ on the surface. The Sr deficiency for 3C-SrIrO_3_ is because the acid treatment is necessary for the removal of the SrCO_3_ impurities during the material synthesis, and simultaneously, the acid treatment also results in some strontium leached from 3C-SrIrO_3_. This result is also in agreement with the previous study showing the easy strontium leaching of 3C-SrIrO_3_ in acid ^16^.

The electrocatalytic result (Supplementary Figure 16) reveals that 3C-SrIrO_3_ affords a current density of 10 mA cm^−2^_geo_ at an overpotential (*η*) of ~270 mV, which is similar to that (270–290 mV) required by the thin films of 3C-SrIrO_3_ reported previsouly^16^. Obviously, 6H-SrIrO_3_ has a higher catalytic activity than 3C-SrIrO_3_ because the former needs a smaller overpotential of ~248 mV to deliver a current density of 10 mA cm^−2^_geo_. The superior catalytic activity of 6H-SrIrO_3_ is further supported by the result that the former exhibits two times mass activity as high as the later (Fig. 3b).

Moreover, both 6H-SrIrO_3_ and 3C-SrIrO_3_ exhibit good catalytic stability for OER in acid, as shown in Fig. 3c. However, after 30 h-long electrocatalysis test, only ~1% of total Sr content (0.9–1.5 Sr layers of 6H-SrIrO_3_) is detected in the acidic solution in the presence of 6H-SrIrO_3_, whereas ~24% of total Sr content is deviated from the stoichiometry of 3C-SrIrO_3_ due to the Sr leaching during electrocatalysis (Fig. 3d). Considering that 3C-SrIrO_3_ has been a Sr-deficient material with an 18% deviation before the electrocatalysis, the 3C-SrIrO_3_ material after electrocatalysis has a total strontium deficiency up to 42%. The result is also consistent with the 30–50% of total Sr content leaching for the thin films of 3C-SrIrO_3_ as oxygen evolution catalysts in acid^16^. Additionally, HRTEM and X-ray photoelectron spectroscopy (XPS) results show that 6H-SrIrO_3_ has good structural stability and keeps high crystallinity (Fig. 3e and Supplementary Figure 17), but 3C-SrIrO_3_ takes place obvious surface amorphourization during electrocatalysis (Fig. 3f). This is further supported by the high-angle annular dark field (HAADF) STEM image (Supplementary Figure 20), which shows that there is not secondary, amorphous IrO_x_ phase generated on the 6H-SrIrO_3_ surface after OER. The N_2_ isotherm data of 6H-SrIrO_3_ (Supplementary Figure 18) reveal that there are not micropores formed after OER. Moreover, the comparison of the 6H-SrIrO_3_’s CV curves before and after OER (Supplementary Figure 19) shows that the CV shape of 6H-SrIrO_3_ after OER is not exactly same as that before OER, but does not change a lot. This suggests that 6H-SrIrO_3_ might undergo a weak surface reconstruction during OER probably thanks to the slight Sr leaching during OER. It should be pointed out that while the better structural stability of 6H-SrIrO_3_ than 3C-SrIrO_3_ has been unambiguously confirmed, the possibility of a very thin amorphous IrO_x_ layer (or a tiny amount of IrO_x_ clusters) on the 6H-SrIrO_3_ surface cannot be completely ruled out at current stage.

The better structural stability of 6H-SrIrO_3_ in comparison with 3C-SrIrO_3_ can be explained by the comparison of their O K-edge X-ray absorption spectra (Supplementary Figure 21). The comparison reveals that there is stronger Ir 5*d*-O 2*p* hybridization or stronger Ir–O covalence for 3C-SrIrO_3_. The stronger covalence results in the easier structural loss of the perovskite phase or surface amorphourization during electrocatalysis, as suggested by the recent studies on perovskite electrocatalysts ^33^.

In order to further assess the stability of 6H-SrIrO_3_, we compared the Ir leaching during OER in the presence of 6H-SrIrO_3_ and IrO_2_. We used inductively coupled plasma atomic emission spectroscopy (ICP-OES) to detect the Ir species in the electrolyte at a current density of 10 mA cm^−2^_geo_ with 6H-SrIrO_3_ or IrO_2_ as the electrocatalyst. The result (Supplementary Figure 22) reveals that a tiny amount of leached Ir species are detected in the electrolytes for both 6H-SrIrO_3_ and IrO_2_ as the electrocatalysts, but the amount of leached Ir species in the presence of 6H-SrIrO_3_ as the electrocatalyst is obviously lower than that in the presence of IrO_2_ as the electrocatalyst. This result further demonstrates that 6H-SrIrO_3_ is a stable electrocatalyst for OER in acid.

In order to determine the Faradaic efficiency during the OER, we compared the amount of O_2_ produced from the OER in the presence of 6H-SrIrO_3_ with the theoretically expected O_2_ amount that can be generated from the OER. The result (Supplementary Figure 23) reveals that the detected O_2_ amount is very close to the expected value during the OER, further demonstrating the about 100% Faradaic efficiency given by 6H-SrIrO_3_.

## Discussion

**Fig. 4:**
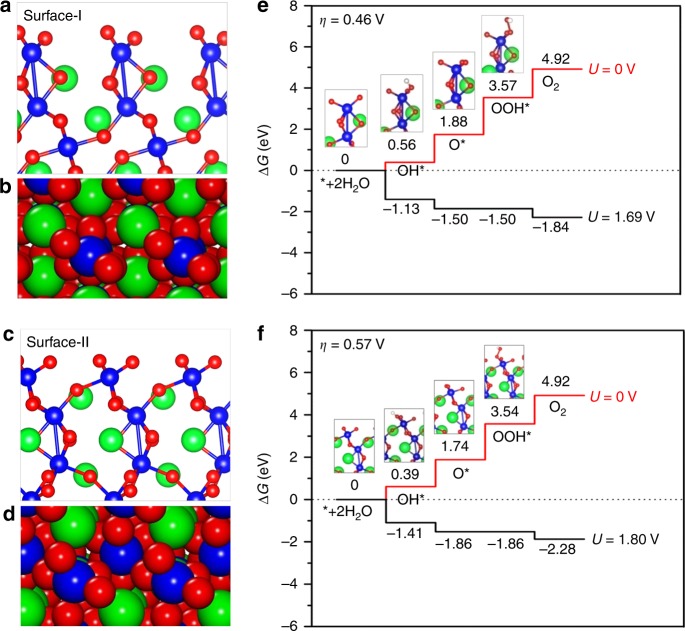
Theoretical understanding of electrocatalytic activity for OER of 6H-SrIrO_3_. **a** Side and **b** top views of the surface-I of 6H-SrIrO_3_; **c** Side and **d** top views of the surface-II of 6H-SrIrO_3_; free-energy diagrams of four elementary reaction steps for the OER on **e** the surface-I and **f** surface-II of 6H-SrIrO_3_ at the different applied potentials. The optimized structures of HO, O, and HOO adsorptions on the surface-I and surface-II are also shown in **e** and **f**

We performed density functional theory (DFT) computations in order to get a better understanding of the electrocatalytic activity of 6H-SrIrO_3_ for OER. We constructed the correlative theoretical models in view of the above experimental results, including the {001} facet of 6H-SrIrO_3_ as the main exposed one and a small amount of surface strontium leached during electrocatalysis. We cleaved the optimized 6H-SrIrO_3_ bulk structure through its (001) plane to obtain two representative surfaces (Fig. 4a–d). The surface-I exposes the Ir atoms in the face-sharing octahedral dimers (Fig. 4a, b), while the surface-II exposes the Ir atoms in the isolated, corner-sharing IrO_6_ octahedra (Fig. 4c, d). On the basis of these surface models, we investigated the electrocatalytic activity for OER of 6H-SrIrO_3_ according to the approach proposed by Rossmeisl et al.^34,35^ In this approach, the OER is suggested to include the four elementary reaction steps and each step involves one proton/one electron-coupled transfer process, as shown below:

1 $${\mathrm{H}}_2{\mathrm{O}} + ^ \ast \to {\mathrm{HO}}^ \ast + {\mathrm{H}}^ + + {{e}}^ -$$

2 $${\mathrm{HO}}^ \ast \to {\mathrm{O}}^ \ast + {\mathrm{H}}^ + + {{e}}^ -$$

3 $${\mathrm{O}}^ \ast + {\mathrm{H}}_2{\mathrm{O}} \to {\mathrm{HOO}}^ \ast + {\mathrm{H}}^ + + {{e}}^ -$$

4 $${\mathrm{HOO}}^ \ast \to ^ \ast + {\mathrm{O}}_2 + {\mathrm{H}}^ + + {{e}}^ -$$

where * and X* (X = HO, O, or HOO) represent a surface active site and an adsorbed X intermediate on the surface, respectively.

As shown in Fig. 4e and f, all of the four elementary reaction steps involved in the OER on both surface-I and surface-II are found to move uphill in free energy when no potential is applied (*U* = 0 V). An additional potential has to be applied to make every step go downhill in free energy, and the minimum additional potentials are determined to be 1.69 and 1.80 V for the surface-I and surface-II, respectively. Considering the equilibrium potential of the OER (*U* = 1.23 V), the minimum theoretical overpotentials (*η*) are calculated to be 0.46 and 0.57 V for the surface-I and surface-II, respectively. These results further demonstrate that the surface-I, comprising the Ir atoms in the face-sharing octahedral dimers, has better catalytic activity for OER than the surface-II with the Ir atoms in the isolated, corner-sharing IrO_6_ octahedra. For comparison, we also explore the OER activities on the (110) surface of IrO_2_ and (010) surface of 3C-SrIrO_3_, and both the sampled surfaces have the similar structural feature to the surface-II, as illustrated in Supplementary Figures 24 and 25. Our computed overpotentials are 0.59 and 0.56 V for IrO_2_ and 3C-SrIrO_3_, respectively, both of which are comparable to that of the surface-II (0.57 V) and larger than that of surface-I (0.46 V). It is worth mentioning that our computed overpotential on the (110) surface of IrO_2_ is also close to the previously reported one^34^. The above calculations imply that the existence of face-sharing octahedral dimers (the main structural feature of surface-I) is mainly responsible for the superior OER activity of 6H-SrIrO_3_, as observed in the above experimental results.

From the Fig. 4e and f, we can also find that the potential-determining step for both surface-I and surface-II is the third elementary reaction step involved in the OER (i.e., O* + H_2_O → HOO* + H^+^ + *e*_¯_) because the maximum Gibbs free-energy difference is between Δ*G*_HOO*_ and Δ*G*_O*_. The facilitated potential-determining step involved in the OER for the surface-I, with respect to the surface-II, can be attributed to the relatively weak O binding (or the relatively low ability of Ir atoms to bind oxygen) on the surface-I, because the surface-I binds oxygen with an O* binding energy of 1.848 eV, while the surface-II binds O* more strongly (Δ*E*_O*_ = 1.711 eV).

When DFT calculations have provided some useful insights into the catalytic active sites and the activity trend for the Ir-based materials from the theoretical computations is consistent with that from our experiments, there is difference between the thermodynamic overpotentials obtained by DFT and the experimental ones. This difference might be caused by multiple factors. First, all the computations are based on thermodynamic considerations. Other important electrocatalytic processes, such as mass and charge transport, cannot be involved in the DFT calculations. Second, the distribution and concentration of active sites on the real material surface are generally very complex, but this complexity cannot be adequately reflected by DFT calculations. Third, at current stage, DFT calculations are not capable of providing atomic information on the more complex catalyst-electrolyte interface, which is crucially important for the electrocatalysis process.

In summary, 6H-SrIrO_3_ has been identified as a highly efficient, low-iridium oxygen evolution electrocatalyst that can work well in acidic media. 6H-SrIrO_3_ is an unusual perovskite oxide electrocatalyst with face-sharing IrO_6_ octahedral dimers. The unique face-sharing IrO_6_ octahedral subunits of 6H-SrIrO_3_ are proven to be important for its remarkable catalytic activity and structural stability. These good properties also make 6H-SrIrO_3_ a promising electrocatalyst for proton exchange membrane (PEM) water electrolysis. The findings in this work might help spark a kind of creativity in the rational design/synthesis of high-performance electrocatalysts for the acidic OER.

## Methods

### Chemicals and reagents

Potassium hexachloroiridate(IV) (K_2_IrCl_6_, 99.99%) was purchased from Aladdin. Strontium nitrate (Sr(NO_3_)_2_), citric acid monohydrate (C_6_H_8_O_7_·H_2_O), ethylene glycol, hydrochloric acid (HCl) and sulfuric acid (H_2_SO_4_) were purchased from Beijing Chemical Factory. Cobalt nitrate hexahydrate (Co(NO_3_)_2_·6H_2_O) was purchased from Shantou Xilong Chemical Factory. Nafion® perfluorinated resin solution (5 wt.% in mixture of lower aliphatic alcohols and water containing 45% water) was purchased from Sigma-Aldrich. All the chemicals and reagents were used without further purification. Highly purified water (>18 MΩ cm resistivity) was provided by a PALL PURELAB Plus system.

### Materials synthesis

For the synthesis of 6H-SrIrO_3_, Sr(NO_3_)_2_ (280 mg) and citric acid (280 mg) were mixed with 5 mL deionized H_2_O to form the solution 1, and K_2_IrCl_6_ (80 mg) was mixed with 4 mL ethylene glycol to form the solution 2. The solution 1 was then stirred into the solution 2 dropwise. The resulting mixture was dried at 150 °C for 12 h to obtain a brown solid product as the precursor, followed by calcination in air at 200 °C (6 h), 300 °C (6 h), 500 °C (3 h), and 700 °C (6 h) in succession. The heating rate was 1.7 °C min^−1^. The obtained solid product containing SrIrO_3_ and SrCO_3_ was treated with 1 M HCl for 6 h to remove the SrCO_3_ impurities, giving the 6H-SrIrO_3_ material. The sequence of synthesis steps was refined through trials and errors.

In order to further assess the stability of 6H-SrIrO_3_ in 1 M HCl solution, we dispersed the as-obtained 6H-SrIrO_3_ powders in 1 M HCl solution for 2 days, and then used ICP-OES to detect the Sr and Ir ions in the solution. The result shows that there is no detectable leached Sr and Ir species in the solution, demonstrating the good stability of 6H-SrIrO_3_ in 1 M HCl solution.

For the synthesis of 3C-SrIrO_3_, the experimental procedures are almost the same as these for the synthesis of 6H-SrIrO_3_, except the amount of citric acid (840 mg) that was used to form the solution 1.

For the synthesis of Co-doped 3C-SrIrO_3_ as a control sample, the experimental procedures are almost the same as these for the synthesis of 6H-SrIrO_3_. The only difference was that a certain amount of cobalt nitrate hexahydrate (14.5 mg) was added to form the solution 2. In the resulting sample, labeled as Co-doped 3C-SrIrO_3_, the Co:Ir atomic ratio is about 1:4.

In order to synthesize the SrIrO_3_ phases (6H-SrIrO_3_ and 3C-SrIrO_3_) without IrO_2_ as the secondary phase, an excessive amount of strontium (Sr:Ir atomic ratio = 8:1) was used during the synthesis in this work. The use of the excessive amount of strontium resulted in the formation of strontium carbonates (SrCO_3_) as the impurities (please see Supplementary Figure 26). The acid treatment with 1 M HCl was employed to remove the SrCO_3_ impurities.

For the synthesis of IrO_2_, the experimental procedures are almost the same as these for the synthesis of 6H-SrIrO_3_. The only difference was that Sr(NO_3_)_2_ was not involved in the synthesis. Supplementary Figure 27 presents XRD patterns of the IrO_2_ samples obtained by calcining the precursor at 300, 500, and 700 °C.

### Characterizations

Powder X-ray diffraction patterns (PXRD) of the samples were collected with an X-ray diffractometer (RIGAKU, Japan, model D/MAX2550 V/PC) with a scan speed of 1° min^−1^ within the diffraction angle range from 10 to 140°. Profile fitting for the PXRD pattern was performed with general structure analysis system (GSAS) program. The scanning electron microscope (SEM) images were obtained with a JEOL JSM 6700F electron microscope. The transmission electron microscope (TEM) images were obtained with a Philips-FEI Tecnai G2S-Twin microscope equipped with a field emission gun operating at 200 kV. Inductively coupled plasma atomic emission spectroscopy (ICP-OES) was performed on a Perkin-Elmer Optima 3300DV ICP spectrometer. The X-ray photoelectron spectroscopy (XPS) was performed on an ESCALAB 250X-ray photoelectron spectrometer with a monochromatic X-ray source (Al K*α hυ* = 1486.6 eV). The O K-edge X-ray absorption near-edge (XANES) spectra were measured in the total electron yield mode in a vacuum chamber (<5 × 10^−8^ Pa). This measurement was performed at the BL12B-a beamline of National Synchrotron Radiation Laboratory (NSRL) in China. The electrical resistivity was investigated with Van Der Pauw method, and the measurement was conducted in a Physical Property Measurement System (PPMS, Quantum Design). High-angle annular dark field scanning transmission electron microscopy (STEM) images were acquired using an aberration-corrected STEM with the model of FEI Titan Cubed Themis G2 300, whose accelerating voltage and electron current were set at 200 kV and around 40 pA, respectively. (Note that because 6H-SrIrO_3_ is very sensitive to the electron beams, high-angle annular dark field STEM images must be obtained at the lowest electron current of 40 pA).

### Electrochemical measurements

All the electrochemical measurements were performed with a three-electrode system by using a CHI instrument (Model 650E). The electrolyte was 0.5 M H_2_SO_4_, and was bubbled with O_2_ gas during the electrochemical measurements. A carbon rod was used as the counter electrode, and a saturated calomel electrode (SCE) was used as the reference electrode. The saturated calomel electrode was calibrated by the reversible hydrogen electrode (RHE), giving their conversion equation: *E*_vs.RHE_ = *E*_vs.SCE_ + 0.241 V. The scan rate was 0.5 mV s^−1^ when linear sweep voltammetry (LSV) was used. The data in this work were compensated by 85% iR-drop. The geometric surface area of the electrode was used to normalize the current density.

For the preparation of working electrodes, the powdered catalyst (7 mg), including 6H-SrIrO_3_, 3C-SrIrO_3_, and IrO_2_, was dispersed in a mixture comprising isopropanol (100 μL) and Nafion solution (10 μL, 5 wt.% in mixture of lower aliphatic alcohols and water). 1 μL of the resulting mixture was then drop-casted onto a glassy carbon electrode with a diameter of 3 mm for drying. The working electrode had a catalyst loading of ca. 0.90 mg cm^−2^.

The electrochemically active surface area (ECSA) of each sample was obtained by determining the double-layer capacitance at non-Faradaic potential range, according to the method reported by Jaramillo et al.^36^ A series of cyclic voltammetry (CV) measurements were performed first at various scan rates (10, 25, 50, 75 and 100 mV s^−1^) in the potential window between 0.83 and 0.93 V vs. RHE. Then, a linear plot was obtained by establishing the relationship between the difference of the anodic and cathodic currents (*i*_a_ − *i*_c_) at 0.88 V vs. RHE and the scan rate. The double-layer capacitance (*C*_dl_) is one half of the slope value of the fitting line. The ECAS can be obtained by dividing *C*_dl_ by a specific capacitance (*C*_s_). A *C*_s_ value of 0.035 mF cm^−2^ was suggested in the previously reported work ^36^.

The reference electrode (i.e., saturated calomel electrode, SCE) was calibrated by the reversible hydrogen electrode (RHE) in 0.5 M H_2_SO_4_. Two Pt electrodes were first cleaned by cycled in 0.5 M H_2_SO_4_ between −2 and 2 V for 2 h, and then used as the working electrode and the counter electrode, respectively. During the calibration, the electrolyte was saturated by H_2_. A series of controlled-potential chronoamperometric curves were carried out around the possible zero current potential (the interconversion between the hydrogen oxidation and hydrogen evolution reaction) with a dwell time of 5 min. The result showed that the potential of zero net current was found at −0.241 V vs. the SCE electrode. And thus, the potentials, measured against SCE, were converted into the potentials vs. RHE by using the equation: *E*_vs.RHE_ = *E*_vs.SCE_ + 0.241 V.

### Computation details

The generalized gradient approximation (GGA) with the Perdew–Burke–Ernzerhof exchange-correlation functional^37^ (including a semi-empirical van der Waals (vdW) correction to account for the dispersion interactions)^38,39^ and a 400-eV cutoff for the plane-wave basis set are employed to perform all the density functional theory (DFT) computations within the frame of Vienna ab initio simulation package (VASP)^40,41^. The projector-augmented plane wave (PAW) is used to describe the electron-ion interactions^42,43^. 5 × 3 × 2 and 5 × 5 × 1 Monkhorst-Pack grid *k*-points are employed for geometric optimization of 6H-SrIrO_3_ and the corresponding slab surfaces, respectively. The density of state (DOS) of the 6H-SrIrO_3_ is computed by using 77 *k*-points. The convergence threshold is set as 10^−4^ eV in energy and 0.05 eV/Å in force. For all the calculations of slab models, the symmetrization is switched off and the dipolar correction is included. Our computed lattice parameters for 6H-SrIrO_3_ are about *a* = 5.554, *b* = 9.584, and *c* = 14.024 Å, all of which are in good agreement with their corresponding experimental values ^28^.

The free energy of H^+^ + *e*_¯_ can be half of formation energy of H_2_ at 298 K and 1 atm. The free energy of the OER is computed by the equation Δ*G* = Δ*E* + ΔZP*E* – *T*Δ*S*. The value of Δ*E* is obtained by the computation of geometrical structures. The values of ΔZP*E* and Δ*S* are determined by employing the computed vibrational frequencies and standard tables for the reactants and products in the gas phase^44^. The entropy for the adsorbed atoms/molecules at the surface active site are assumed to be zero. The temperature dependence of the enthalpy is neglected in the calculations. Moreover, an external bias *U* is imposed on each step by including a *−eU* term in the computation of reaction free energy. Consequently, the reaction free energy of each step can be expressed as follows:

5 $$\Delta G_{1} = E\left( {\mathrm{HO}}^{\ast}\right) - E\left( {\,\!}^{\ast}\right) - E_{{\mathrm{H2O}} + }1/2 E_{{\mathrm{H2}}} + \left( {\Delta {\mathrm{ZPE}} - T \Delta S} \right)_{{\mathrm{A}}}-eU$$

6 $$\Delta G_{2} = E\left( {\mathrm{O}}^ {*} \right) - E\left( {\mathrm{HO}} ^{\ast} \right) + 1/2 E_{{\mathrm{H2}}} + \left( {\Delta {\mathrm{ZPE}} - T\Delta S} \right)_{{\mathrm{B}}}-eU$$

7 $$\Delta G_{3} = E\left( {\mathrm{HOO}}^{\ast} \right) - E\left( {\mathrm{O}}^{\ast} \right) - E_{{\mathrm{H2O}}} + 1/2 E_{{\mathrm{H2}}} + \left( {\Delta {\mathrm{ZPE}} - T\Delta S} \right)_{{\mathrm{C}}}-eU$$

8 $$\Delta G_{4} = E\left( {\,\!}^{\ast}\right) - E\left( {\mathrm{HOO}}^{\ast} \right) + E_{{\mathrm{O2}}} + 1/2 E_{{\mathrm{H2}}} + \left( {\Delta {\mathrm{ZPE}} - T\Delta S} \right)_{{\mathrm{D}}}-eU$$

where *E*(*), *E*(HO*), *E*(O*), and *E*(HOO*) are the computed DFT energies of the pure surface and the adsorbed surfaces with HO*, O*,and HOO*, respectively. *E*_H2O_, *E*_H2_, and *E*_O2_ are the computed energies for the sole H_2_O, H_2_ and O_2_ molecules, respectively. For the total reaction H_2_O → 1/2O_2_ + H_2_, the free-energy change is fixed at the experimental value of 2.46 eV per water molecule. When forming one molecule of O_2_ in the reaction step, the reaction free energy can be expressed as ∆*G*_(2H2O→O2+2H2)_ = 4.92 eV = *E*_O2_ + 2*E*_H2_ − 2*E*_H2O_ + (∆ZPE − *T∆S*)_(2H2O→O2+2H2)_. The reaction overpotential can be obtained by evaluating the difference between the minimum voltage needed for the OER and the corresponding voltage needed for changing all the free-energy steps into downhill. The correlative theoretical models are constructed based on the experimental results, including the {001} facet of 6H-SrIrO_3_ as the main exposed one and a small amount of surface strontium leached during electrocatalysis. The dissolution of SrIrO_3_ to form Sr^2+^ can be written as SrIrO_3_ + 2 H^+^ → IrO_2_ + Sr^2+^ + H_2_O, and in this situation, Sr atoms and the related O atoms in the uppest layer for these structural models are discarded from a stoichiometric SrIrO_3_ (001) surface. All the theoretical models have the thickness of six layers. During the computational process, the upper two layers in the model are fully relaxed without any symmetry or directional restrictions, while the remaining four layers are kept frozen.

The ELF was extensively employed to understand the localized bonding character in the crystal structures^26,27^. The ELF can be described in the form of a contour plot in real space with values ranging from 0 to 1. The region close to 1 indicates the presence of strong covalent electrons or lone-pair electrons. While the region near to 0.5 represents homogenous electron gas or the presence of metallic bonds, the region close to 0 means a low electron density area. The ELF is different from the molecular orbital theory that can display the orbital orientation. In our work, we employed the ELF method to confirm the existence of Ir–Ir metallic bond in the face-shared IrO_6_ dimers. As presented in Fig. 1d, the correlative ELF values with a range of 0.4 to 0.5 distribute between the two neighboring Ir atoms in face-sharing IrO_6_ dimers, indicating that Ir–Ir bond is metallic in character.

## Electronic supplementary material

Supplementary Information

Peer Review File

## Data Availability

The authors declare that the data supporting the findings of this study are available within the paper and its supplementary information files.
