# Supplementary material for: Efficient oxygen evolution electrocatalysis in acid by a perovskite with face-sharing IrO6 octahedral dimers
Source: Nat Commun. 2018 Dec 7;9:5236. doi: 10.1038/s41467-018-07678-w (PMC6286314; doi:10.1038/s41467-018-07678-w)
Supplement: Supplementary file 1 — Supplementary Information [file 41467_2018_7678_MOESM1_ESM.pdf]

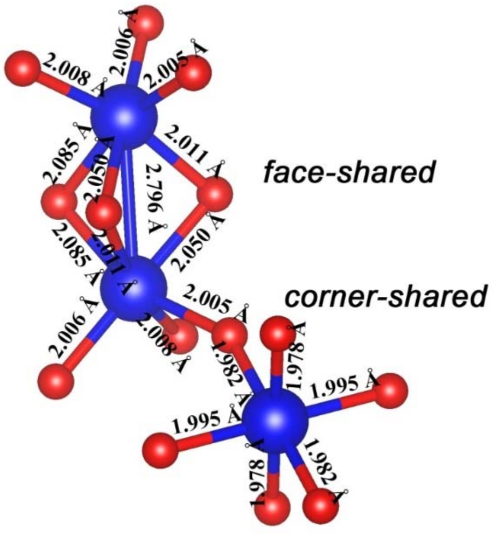

Supplementary Figure 1. Local structure and bond lengths of 6H-$SrIrO_3$. Local ball-and-stick model of 6H-$SrIrO_3$, in which Ir-Ir and Ir-O bond lengths are presented.

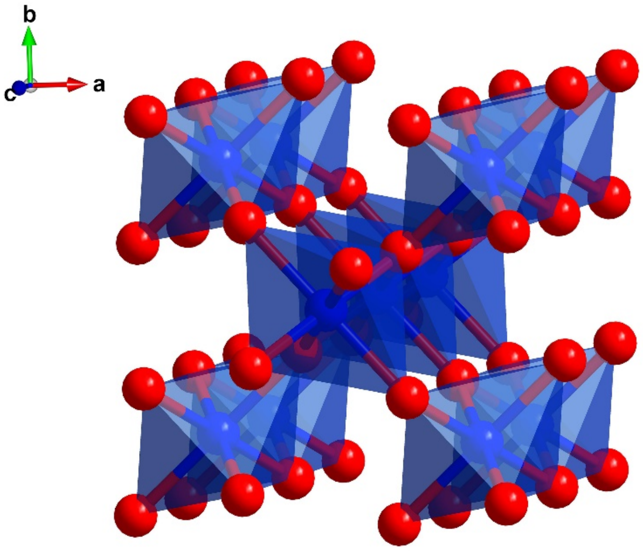

Supplementary Figure 2. Crystal structure of IrO_2. The blue and red balls represent the Ir and O atoms, respectively. There are corner-sharing and edge-sharing IrO_6 octahedra in IrO_2.

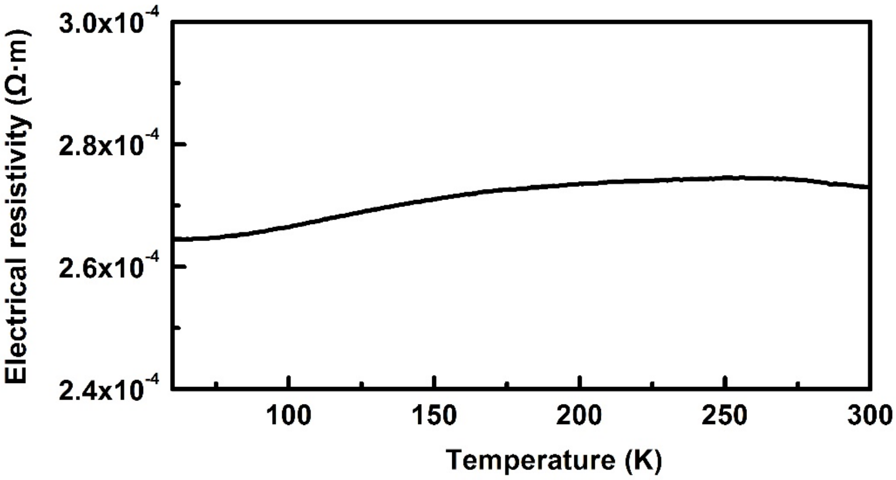

Supplementary Figure 3. Electrical resistivity of 6H-$SrIrO_3$. Temperature dependence of the resistivity for 6H-$SrIrO_3$.

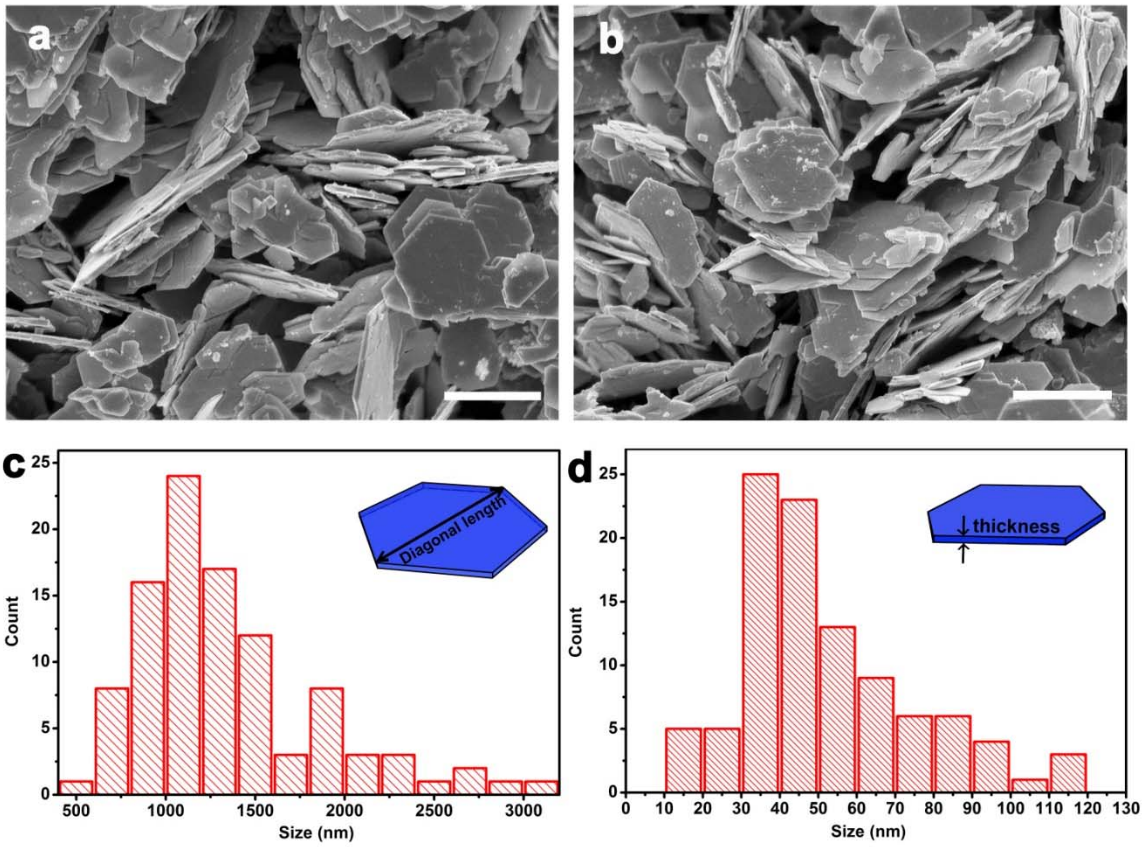

Supplementary Figure 4. Morphology of 6H-$SrIrO_3$. a and **b**, Additional SEM images of 6H-$SrIrO_3$. **c**, Lateral size distribution and **d**, thickness distribution of plate-like 6H-$SrIrO_3$ particles. Scale bars, 2 μm.

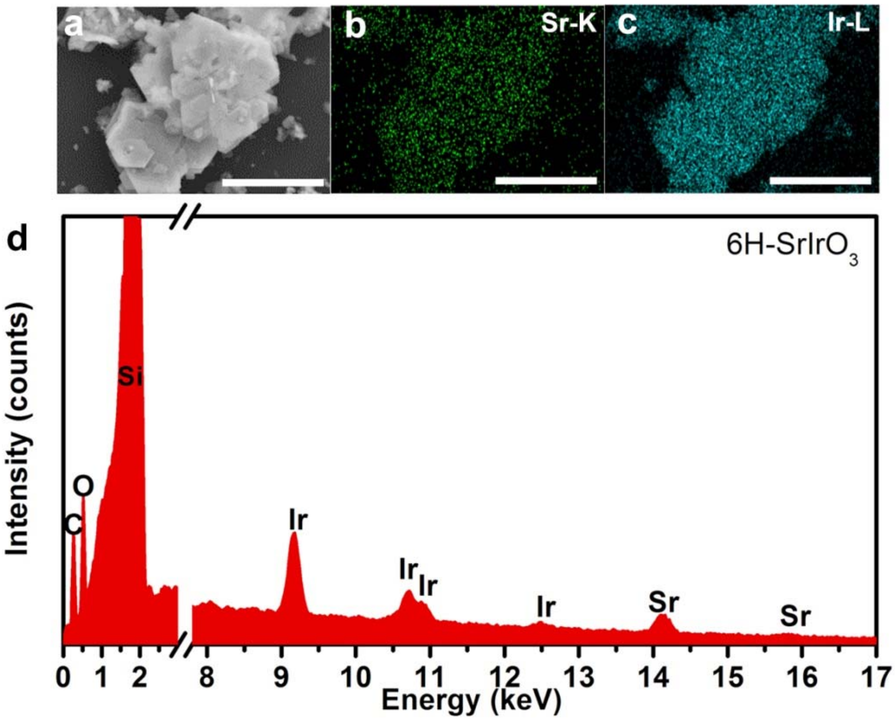

Supplementary Figure 5. Elemental distribution of 6H-$SrIrO_3$. **a**, SEM image, **b** and **c**, the corresponding elemental mapping images and **d**, energy dispersive X-ray spectroscopy (EDS) of 6H-$SrIrO_3$. In supplementary Figure 5d, the Si peak comes from the silicon plate as sample holder. The atomic ratio of Sr:Ir is about 1:1. Scale bars, 10 μm.

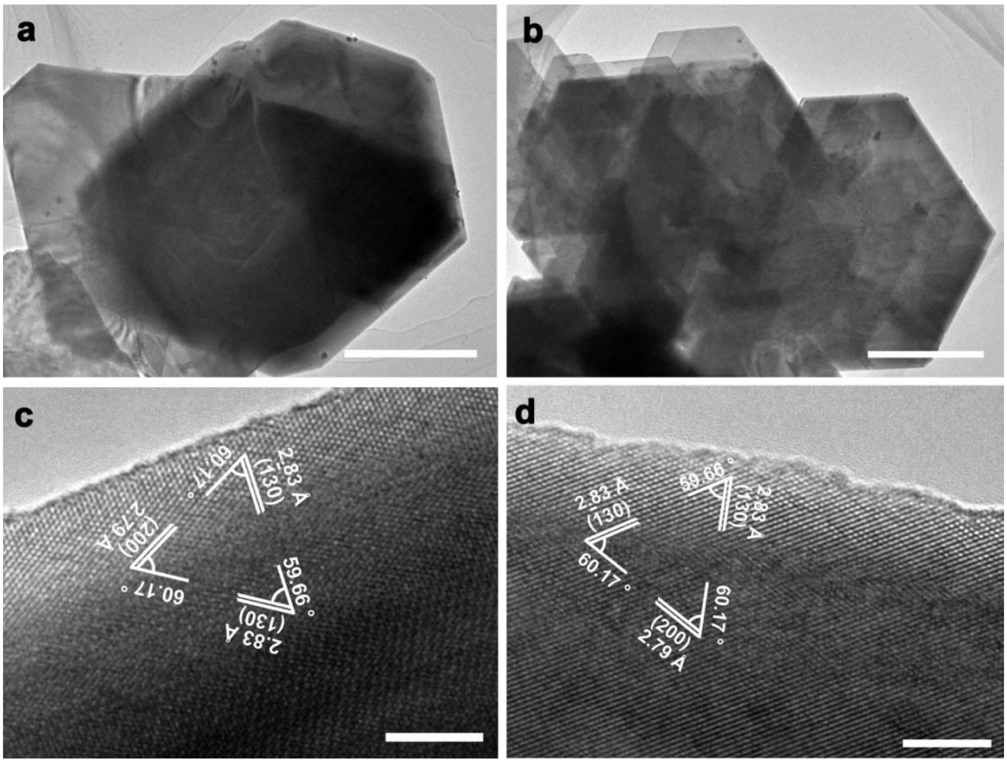

Supplementary Figure 6. TEM characterizations of 6H-$SrIrO_3$. a and **b**, Additional TEM images of 6H-$SrIrO_3$. Scale bars, 500 nm. **c** and **d**, Additional HRTEM images of 6H-$SrIrO_3$. Scale bars, 5 nm.

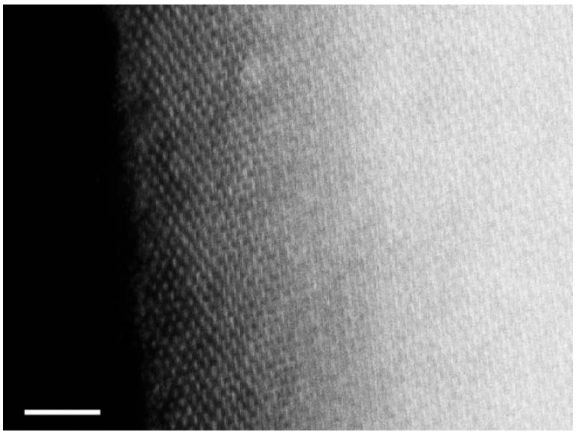

Supplementary Figure 7. HAADF-STEM characterization of 6H-$SrIrO_3$. High-angle annular dark field (HAADF) STEM image of the edge of a 6H-$SrIrO_3$ particle. Scale bar, 2 nm.

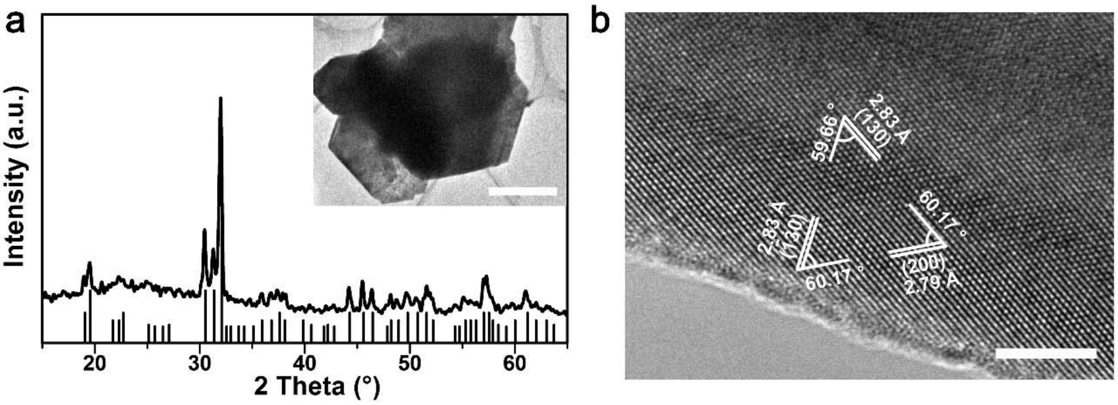

Supplementary Figure 8. Structural characterizations of 6H-$SrIrO_3$ after acid treatment. a, XRD pattern with a TEM image as the inset. Scale bar, 1 μm. **b**, HRTEM image of the 6H-$SrIrO_3$ sample, which was immersed in 0.5 M H_2SO_4 solution for 48 days. Scale bar, 5 nm.

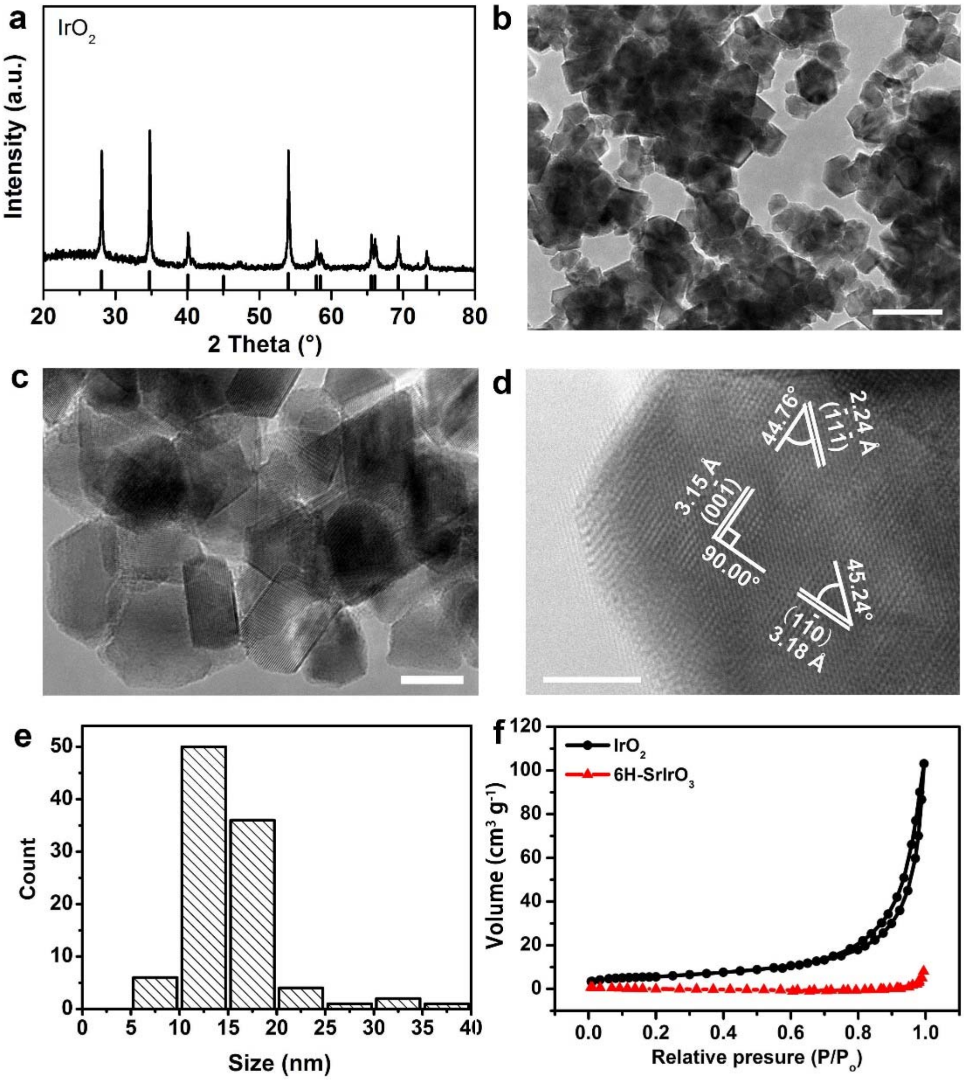

Supplementary Figure 9. Structural characterizations of IrO_2. a, XRD pattern, **b**,**c** TEM images, **d**, HRTEM image, **e**, particle size distribution and **f**, N_2 adsorption–desorption isotherms of IrO_2 nanoparticles. In Supplementary Figure 9f, the N_2 adsorption–desorption isotherms of 6H-$SrIrO_3$ are also provided for comparison. The results reveal that the material is composed of rutile IrO_2 nanoparticles with a particle size of 10-20 nm. Scale bars, 50 nm in **b**, 10 nm in **c**, and 5 nm in **d**.

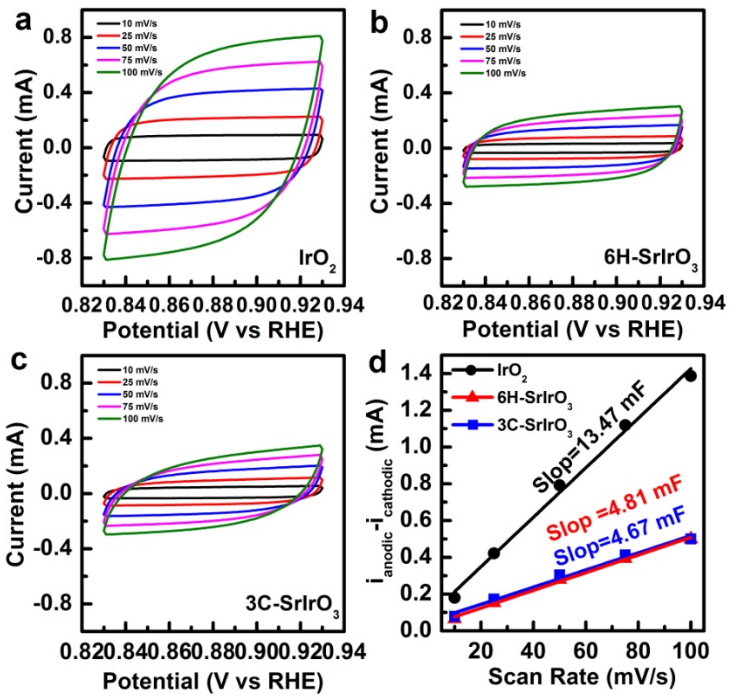

Supplementary Figure 10. a-c, **Electrochemically active surface areas of Ir-based oxide samples.** Cyclic voltammograms with IrO_2, 6H-$SrIrO_3$ and 3C-$SrIrO_3$ measured in a non-Faradaic region of the voltammogram at different scan rates. **d**, The difference in current density between the anodic and cathodic sweeps *versus* scan rate; the slope of the fitting line is used for determination of the double-layer capacitance (C_{dl}).

The electrochemically active surface areas (ECSAs) of IrO_2, 6H-$SrIrO_3$ and 3C-$SrIrO_3$ are estimated to be 192.5, 68.7 and 66.7 cm^2, respectively. The result shows that the ECSA of 6H-$SrIrO_3$ is about 3 times lower than that of IrO_2.

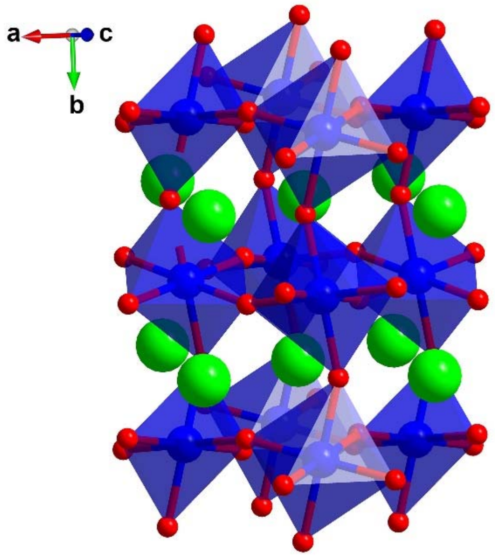

Supplementary Figure 11. Crystal structure of 3C-$SrIrO_3$. The blue, green and red balls represent the Ir, Sr and O atoms, respectively. In its structure, all the IrO_6 octahedra are conner-shared.

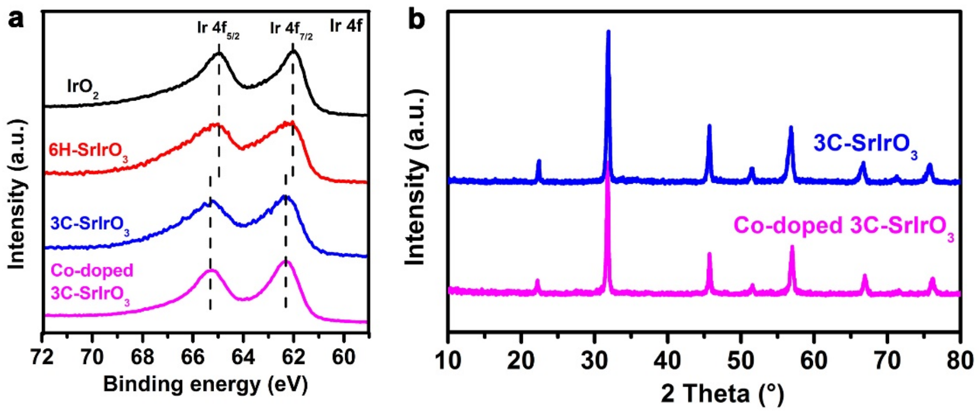

Supplementary Figure 12. XPS and XRD characterizations of several Ir-based oxide samples. a, Ir4f XPS spectra of IrO_2, 6H-$SrIrO_3$, 3C-$SrIrO_3$ and Co-doped 3C-$SrIrO_3$. **b**, XRD patterns of 3C-$SrIrO_3$ and Co-doped 3C-$SrIrO_3$.

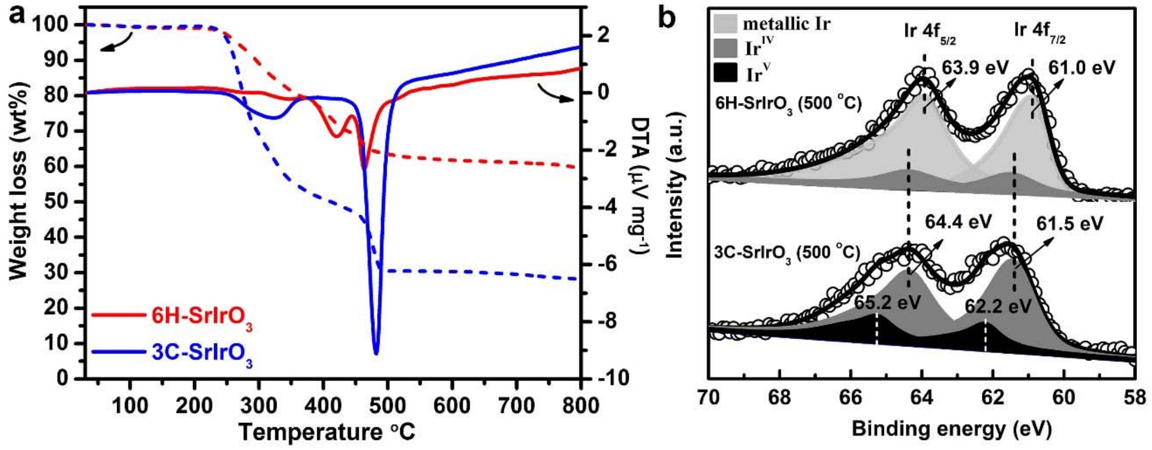

Supplementary Figure 13. TG-DTA and XPS characterizations. **a**, Thermogravimetric (TG) and differential thermal analysis (DTA) in air of the precursors of 6H-$SrIrO_3$ and 3C-$SrIrO_3$. **b**, Ir4f XPS spectra of two samples that are obtianed by calcining the precursors of 6H-$SrIrO_3$ and 3C-$SrIrO_3$ at 500 ^{o}C.

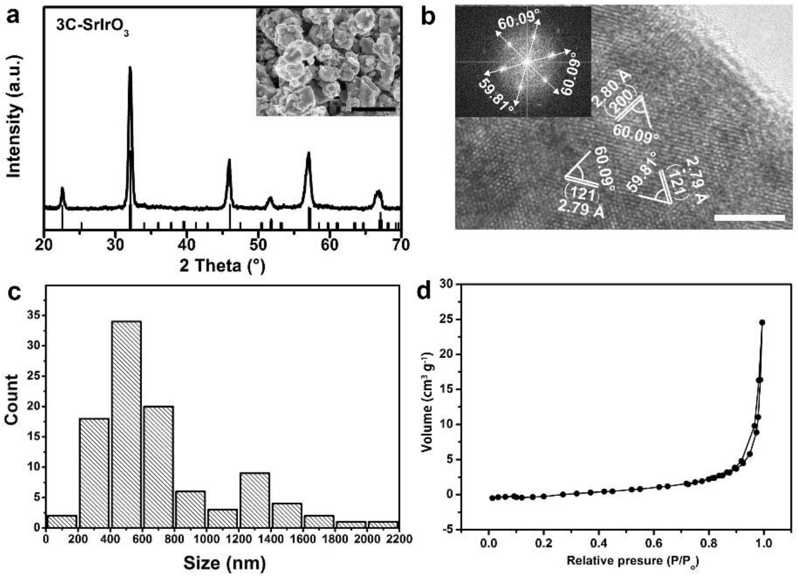

Supplementary Figure 14. Structural characterizations of 3C-SrIrO$_3$. a, XRD pattern of 3C-SrIrO$_3$, with a SEM image shown in the inset. Scale bar, 2 μm. **b**, HRTEM image of 3C-SrIrO$_3$, with the corresponding fast Fourier transform image in the inset. Scale bar, 5 nm. **c**, Particle size distribution and **d**, N_2 adsorption–desorption isotherms of 3C-SrIrO$_3$.

3C-SrIrO$_3$ adopts a well-known pseudo-cubic structure. Its successful synthesis is confirmed by the XRD result (Supplementary Figure 14a). 3C-SrIrO$_3$ contains of micron-sized particles, as shown in the SEM image (Supplementary Figure 14a, inset). In its HRTEM image (Supplementary Figure 14b), three sets of lattice fringes are observed, giving interplanar distances of 2.80, 2.79 and 2.79 Å, corresponding to the (200) , (121) and ($\bar{1}$21) crystallographic planes of 3C-SrIrO$_3$. The observed angles are matched with the theoretical values. The angle between the (200) and ($\bar{1}$21) facets is 60.09°; the angle between the ($\bar{1}$21) and (121) facets is 59.81°; and the angle between the (121) and (200) facets is 59.81°. The observed interplanar distances and the angles between them are calculated from the Fast Fourier Transform (FFT) analyses (Supplementary Figure 14b, inset). Additionally, it should be pointed out that the lattice fringes in the HRTEM image are not very clear and there are some amorphous areas. Moreover, the 3C-SrIrO$_3$ material is a Sr-deficient material with a Sr:Ir atomic ratio of 0.82:1 (Supplementary Figure 15). These combined results indicate that the 3C-SrIrO$_3$ material has a certain amount of IrO_x on the surface because of the necessary acid treatment process resulting in some strontium leached during the material synthesis. Figure Supplementary 14d reveals that 3C-SrIrO$_3$ is a nonporous material.

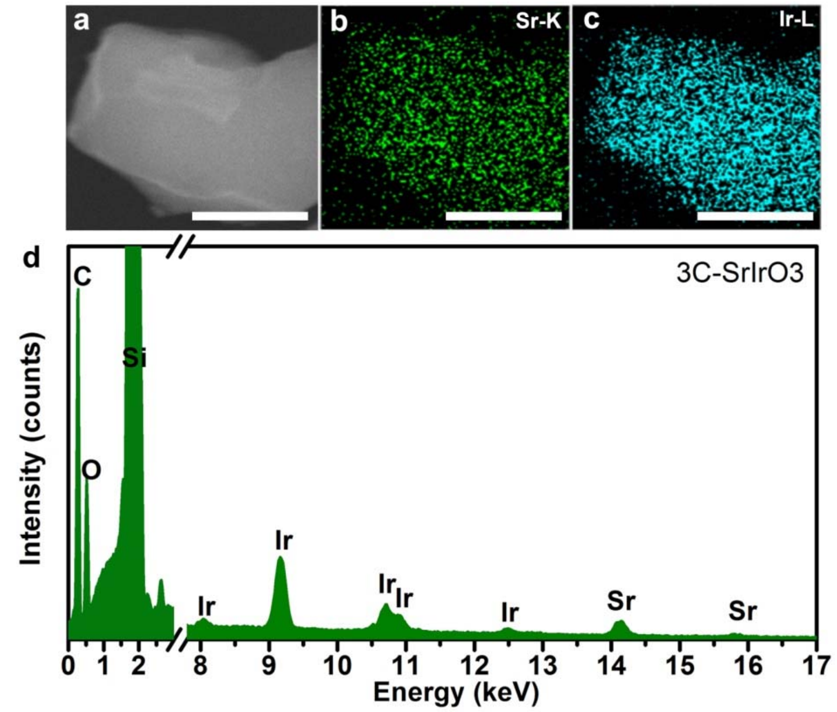

Supplementary Figure 15. Elemental distribution of 3C-$SrIrO_3$. **a**, SEM image, **b** and **c**, the corresponding elemental mapping images and **d**, energy dispersive X-ray spectroscopy (EDS) of 3C-$SrIrO_3$. In Supplementary Figure 15d, the Si peak comes from the silicon plate as sample holder. The atomic ratio of Sr:Ir is about 0.82:1. Scale bars, 500 nm.

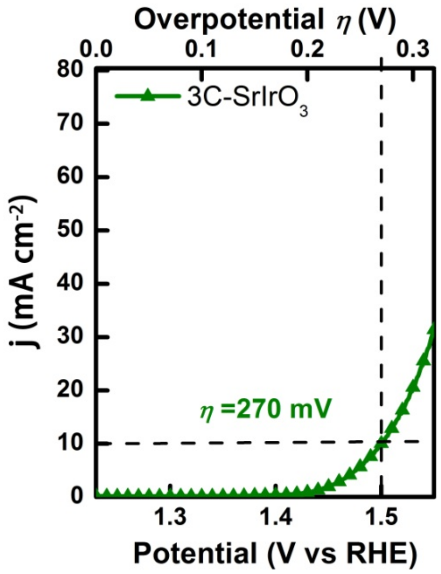

Supplementary Figure 16. Catalytic activity of 3C-$SrIrO_3$. Polarization curve of 3C-$SrIrO_3$ in 0.5 M H_2SO_4 solution with 85% *iR*-compensation. The current densities are normalized by the geometric area.

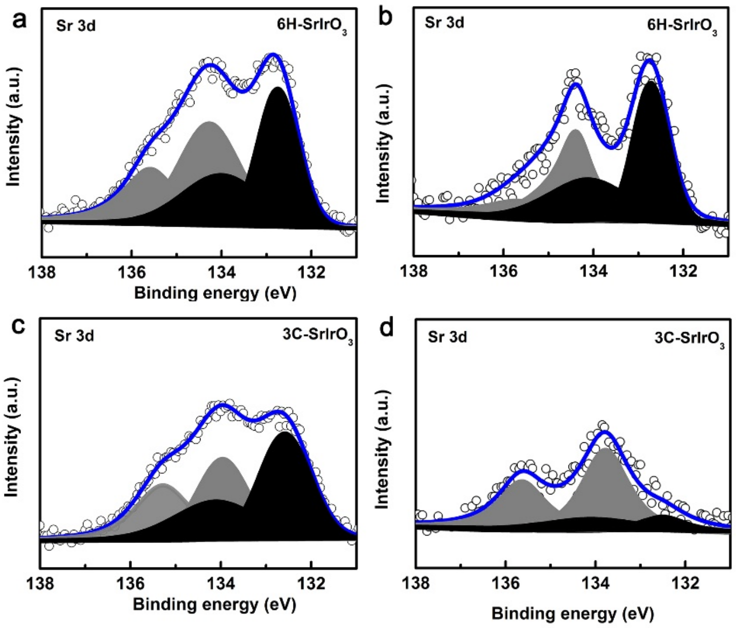

Supplementary Figure 17. Comparison of XPS results of 6H-$SrIrO_3$ and 3C-$SrIrO_3$. Sr3d XPS spectra of 6H-$SrIrO_3$ **a**, before and **b**, after 30 hours of testing. Sr3d XPS Spectra of 3C-$SrIrO_3$ **c**, before and **d**, after 30 hours of testing. Circles: experimental data; blue lines: the fit sum of the doublets, shown in black and gray.

X-ray photoelectron spectroscopy (XPS) was employed to study the local structure of Sr species for 6H-$SrIrO_3$ and 3C-$SrIrO_3$ before and after 30 hours of electrocatalysis testing. The Sr3d XPS spectrum of Sr-containing perovskite is well known to be sensitive to the surface structural rearrangement of the material.[1-3] As shown in Supplementary Figure 17a and 17c, the XPS spectra of 6H-$SrIrO_3$ and 3C-$SrIrO_3$ before the electrocatalysis are similar, and their Sr3d peaks can be fitted with two doublets. The first doublet, shown in black, is assigned to the lattice Sr (or bulk Sr), and the second doublet, shown in grey, is attributed to the surface Sr. Comparison of the Sr3d XPS spectra of 6H-$SrIrO_3$ (Supplementary Figure 17a and 17b) shows that the first doublet keep unchanged, and the second doublet decrease to a certain extent. This result demonstrates that 6H-$SrIrO_3$ can keep its bulk structure intact during electrocatalysis, with a small amount of surface Sr leaching. Comparison of the Sr3d XPS spectra of 3C-$SrIrO_3$ (Supplementary Figure 17c and 17d) shows that the first doublet almost disappears, and the second doublet decreases significantly. This result, in agreement with other characterization results (Figure 3), reveals that 3C-$SrIrO_3$ loses a large amount of Sr and undergoes surface amorphorization during electrocatalysis.

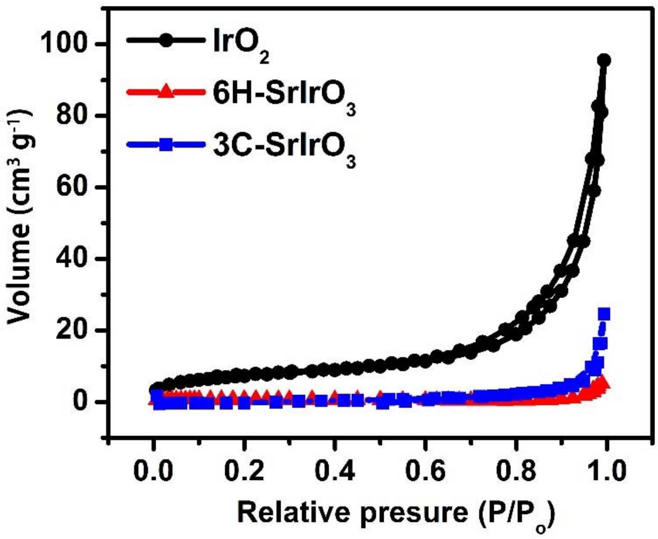

Supplementary Figure 18. N_2 adsorption–desorption characterizations. N_2 adsorption–desorption isotherms of 6H-$SrIrO_3$, 3C-$SrIrO_3$ and IrO_2 after the OER. The results show that there are not micropores for these three materials after OER.[4]

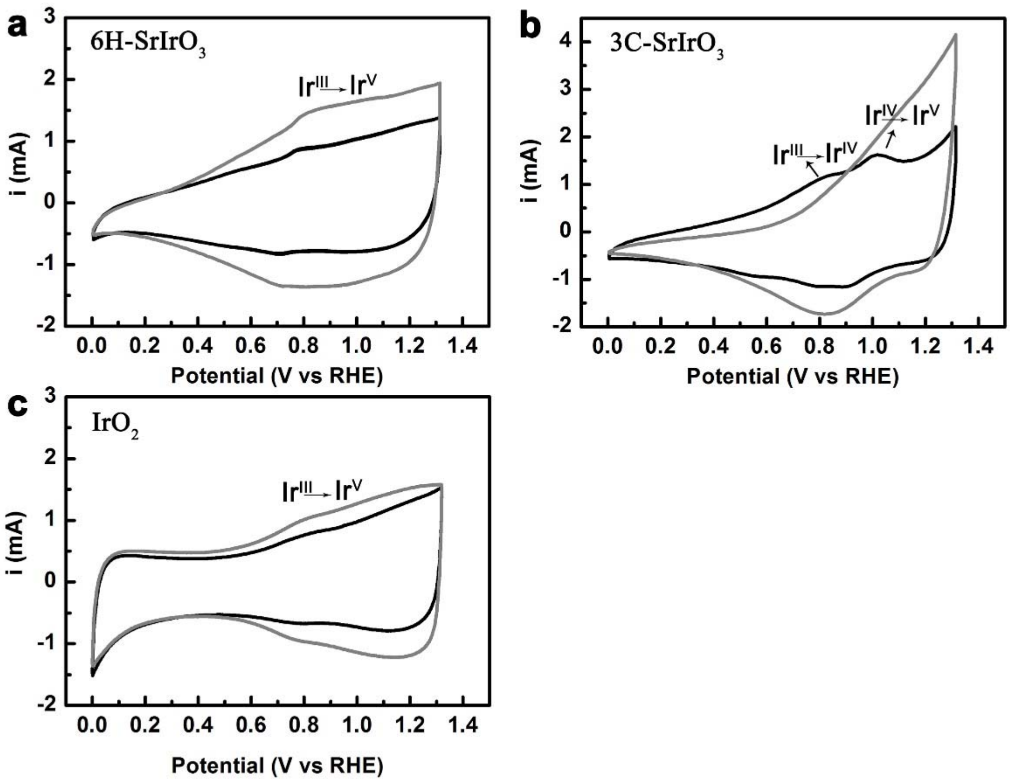

Supplementary Figure 19. CV comparison before and after electrocatalysis. Cyclic voltammograms (200 mV s^{-1}) recorded before (black line) and after (gray line) the OER for **a**, 6H-$SrIrO_3$, **b**, 3C-$SrIrO_3$ and **c**, IrO_2.

As shown in Supplementary Figure 19, the CV curves are not complete overlap before and after OER for all the three samples. Different from that of 3C-$SrIrO_3$, the CV shapes of 6H-$SrIrO_3$ and IrO_2 do not change obviously after OER, indicating that the later two materials do not undergo significant variation in surface structure. The slight increase in the area of CV curve for 6H-$SrIrO_3$ after OER might be due to the slight surface Sr leaching during OER.

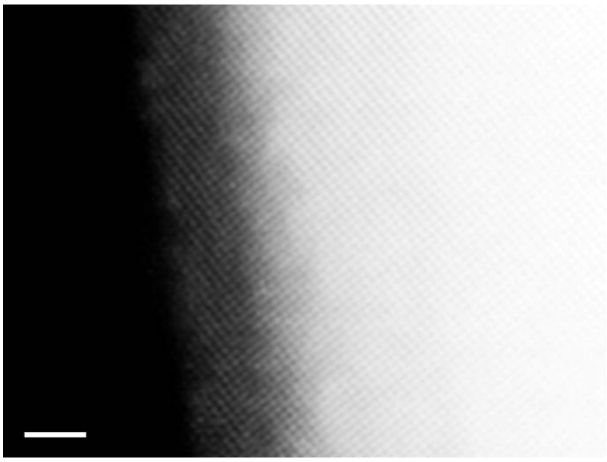

Supplementary Figure 20. HAADF-STEM characterization of 6H-$SrIrO_3$ after OER. High-angle annular dark field (HAADF) STEM image of the edge of a 6H-$SrIrO_3$ particle after OER. Scale bar, 2 nm.

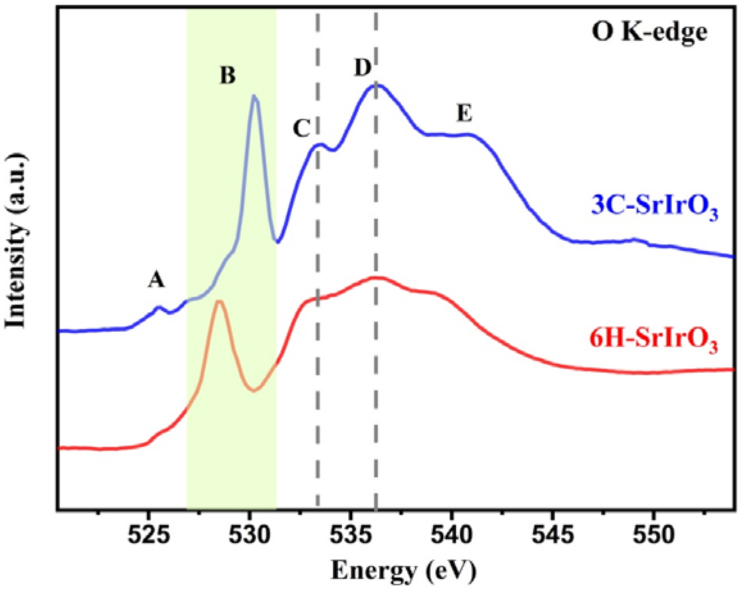

Supplementary Figure 21. XAS characterization. O K-edge X-ray absorption spectra of 3C-$SrIrO_3$ and 6H-$SrIrO_3$.

There are five peaks in the O K-edge X-ray absorption spectra (XAS) of 3C-$SrIrO_3$ and 6H-$SrIrO_3$. The pre-edge peak A at 525.5 eV is assigned to the charge-transfer band (or hole doping). The presence of this peak is due to the strong covalent interactions of Ir-O bonds. The peak B at 528-531 eV can be attributed to the overlapping bands between Ir 5*d* t_{2g} and O 2*p* and the peak C at 532.4-533.7 eV can be assigned to the hybridization between Ir 5*d* e_g and O 2*p*. While the peak D stems from the hybridization between Sr 4*d* and O 2*p*, the Peak E at 538-545 eV is originated from the physisorbed and chemisorbed water molecules. Comparison of the O K-edge X-ray absorption spectra that the peaks B and C for 3C-$SrIrO_3$ appear at higher energy than those for 6H-$SrIrO_3$, indicating that there is stronger hybridization of Ir 5*d*-O 2*p* or stronger Ir-O covalence for 3C-$SrIrO_3$. Additionally, the position of the peak D is almost the same for both 3C-$SrIrO_3$ and 6H-$SrIrO_3$, indicating that their different crystal structure has no effect on the covalence of Sr-O bond.

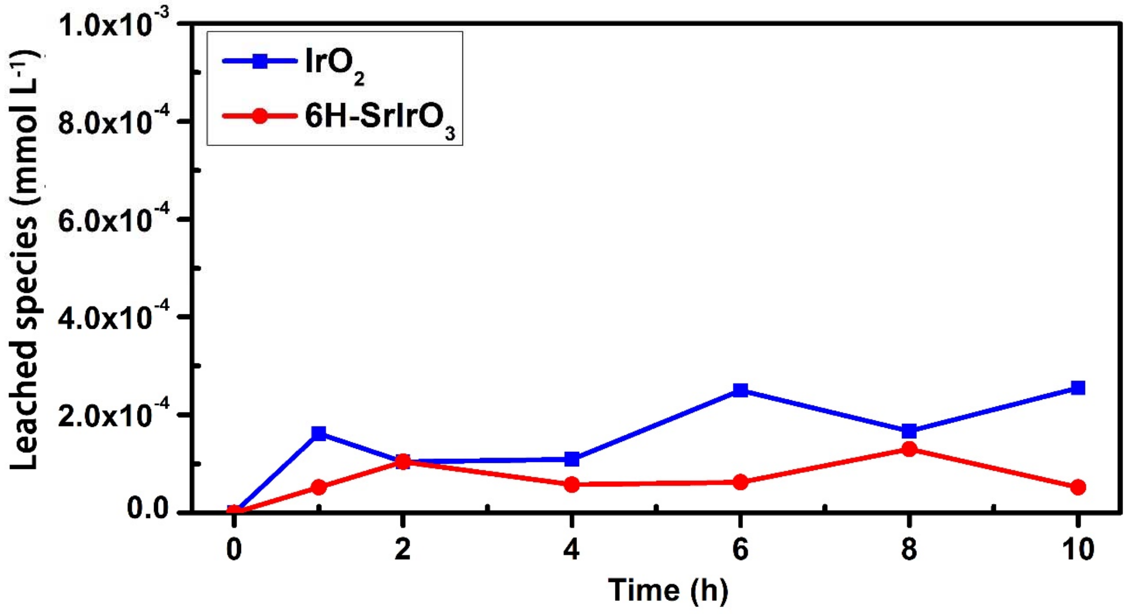

Supplementary Figure 22. Stability comparison between 6H-$SrIrO_3$ and IrO_2. Ir leaching with 6H-$SrIrO_3$ and IrO_2 as the electrocatalyst during oxygen evolution reaction at a current density of 10 mA cm^{-2}.

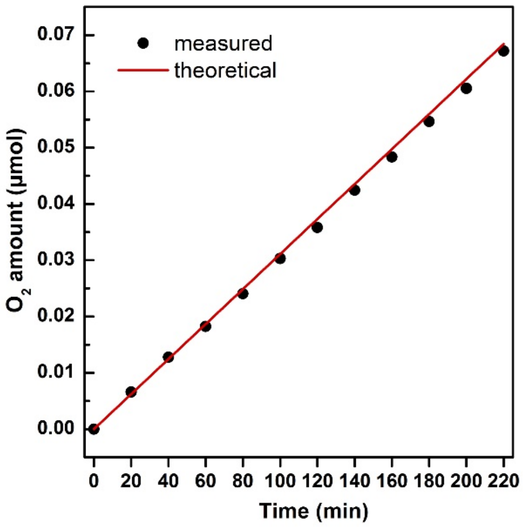

Supplementary Figure 23. Faradic Efficiency during OER. Electrocatalytic efficiency of O_2 production over 6H-$SrIrO_3$ at a current density of *ca.* 20 mA cm^{-2}, measured for 220 min.

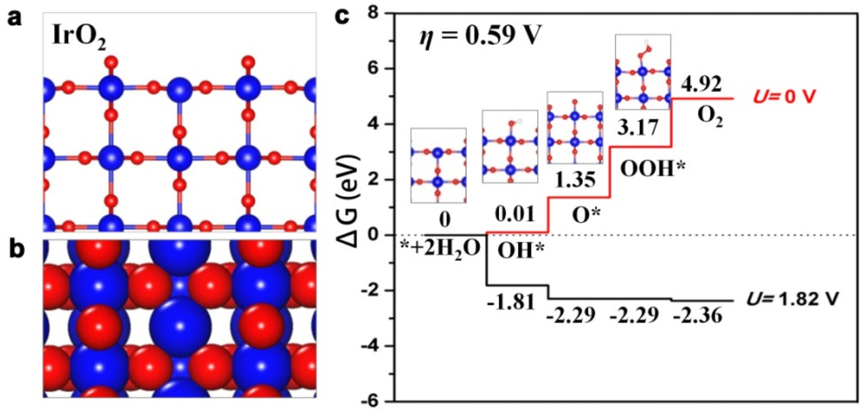

Supplementary Figure 24. Theoretical results for IrO_2. a, Side and **b**, top views of the (110) surface of IrO_2. **c**, Free-energy diagram of four elementary reaction steps for the OER at the different applied potentials. The optimized structures of HO, O and HOO adsorptions on the surface are also shown in Supplementary Figure 24c.

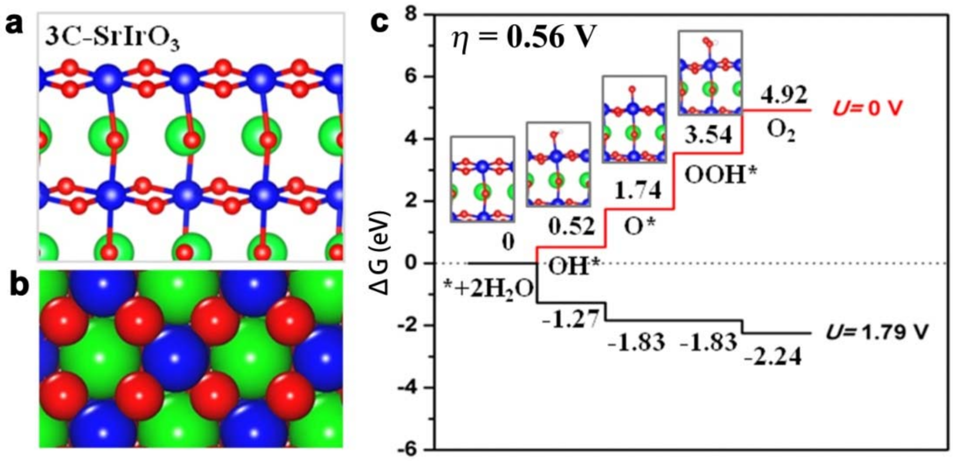

Supplementary Figure 25. Theoretical results for 3C-SrIrO$_3$. **a**, Side and **b**, top views of the (010) surface of 3C-SrIrO$_3$; **c**, Free-energy diagram of four elementary reaction steps for the OER at the different applied potentials. The optimized structures of HO, O and HOO adsorptions on the surface are also shown in Supplementary Figure 25c.

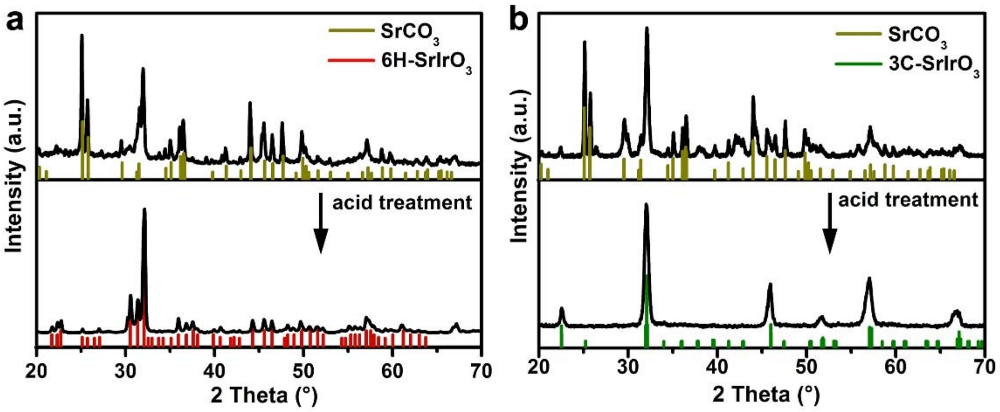

Supplementary Figure 26. XRD characterizations of the samples before and after acid treatment. a and **b**, Comparison of the XRD patterns of the samples before and after acid treatment during the synthesis of 6H-$SrIrO_3$ and 3C-$SrIrO_3$.

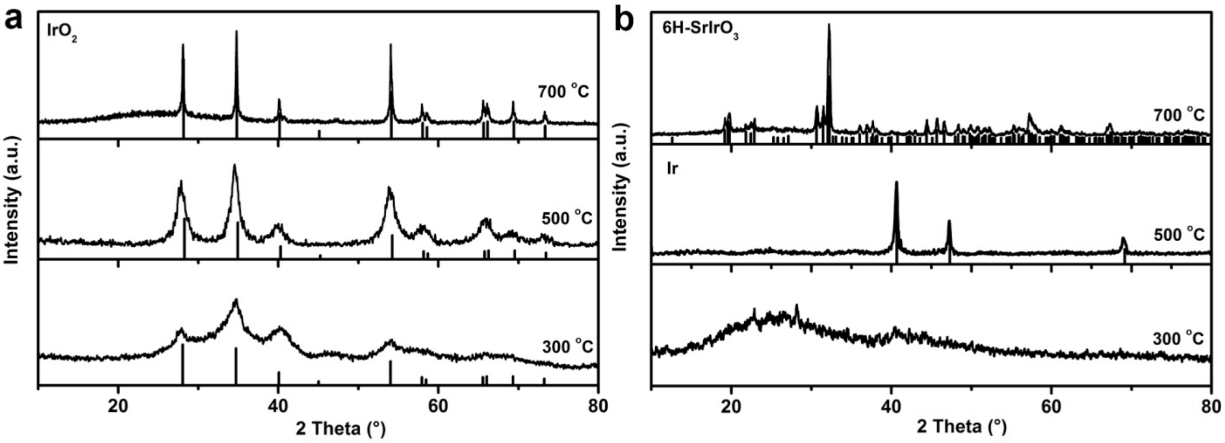

Supplementary Figure 27. XRD characterizations of the samples obtained at different temperatures. **a** and **b,** XRD patterns of the samples obtained by calcining the precursors of IrO_2 and 6H-$SrIrO_3$ at 300, 500 or 700 °C.

As shown in the supplementary figure 27a, IrO_2 starts crystallization at a very low temperature of 300 °C. And with the increase in the reaction temperature, the degree of crystanillity of IrO_2 increase. This can be reflected by the sharper and stronger XRD peaks of the sample at higher temperature. By comparing the synthesis of IrO_2 and 6H-$SrIrO_3$, we can find that although their experimental procedures are almost the same in our work, the crystallization processes are totally different for IrO_2 and 6H-$SrIrO_3$. IrO_2 can crystallize at a very low temperature of 300 ºC, but 6H-$SrIrO_3$ crystallizes until the temperature increase up to 700 ºC (Supplementary Figure 27b). This might be the reason why the experimental procedures are almost the same for the synthesis of IrO_2 and 6H-$SrIrO_3$ in our work, but their areas/sizes are different.

Supplementary Table 1. Crystallographic information for 6H-$SrIrO_3$ according to the PXRD profile fitting results.

Chemical name	Strontium Iridium(IV) Oxide
Chemical formula	$SrIrO_3$
Crystal system	monoclinic
Space group	C2/c
Chemical formula weight (g/mol)	327.84
Cell length a (Å)	5.6113(2)
Cell length b (Å)	9.6335(3)
Cell length c (Å)	14.1863(5)
Cell angle α (°)	90.0
Cell angle β (°)	93.2
Cell angle γ (°)	90.0
Cell volume (Å^3)	765.66(5)
Cell formula units Z	12
Reduced χ^2	10.34
Goodness of fit	3.22
wR_p	0.11
R_p	0.08

Supplementary Table 2. Comparison of BET surface areas and OER activities of 6H-$SrIrO_3$ with some recently reported Ir-based oxide electrocatalysts in acid.

Catalyst	BET surface area	η at 10 mA/ cm^2_{geo}	Reference
**6H-$SrIrO_3$**	**0.3 m^2/g**	**248** mV	**This work**
**IrO_2**	**19.8 m^2/g**	**300** mV	**This work**
**3C-$SrIrO_3$**	**0.3 m^2/g**	**270** mV	**This work**
**3C-$SrIrO_3$ (or IrO_x/ $SrIrO_3$)**	--	270-290 mV	*Science* **2016**,353, 1011
**La_2LiIrO_6**	1.7 m^2/g	~300 mV	*Nat. Energy* **2016**, 2, 16189
**Pb-Ir pyrochlore**	14.8 m^2/g	340-370 mV	*Sci. Rep.* **2016**, 6, 38429
**Bi-Ir pyrochlore**	46 m^2/g	340-370 mV	*Chem. Mater.* **2012**, *24*, 4192
**Y-Ir pyrochlore**	21 m^2/g	>370 mV	*Chem. Mater.* **2017**, 29, 5182
**Bi-Ir pyrochlore**	30 m^2/g	>370 mV	*Chem. Mater.* **2017**, 29, 5182
**Pb-Ir pyrochlore**	24 m^2/g	>370 mV	*Chem. Mater.* **2017**, 29, 5182
**BiY−Ir pyrochlore**	28 m^2/g	>370 mV	*Chem. Mater.* **2017**, 29, 5182
**BiPb−Ir pyrochlore**	38 m^2/g	>370 mV	*Chem. Mater.* **2017**, 29, 5182
**YPb−Ir pyrochlore**	11 m^2/g	>370 mV	*Chem. Mater.* **2017**, 29, 5182
**$W_{0.7}Ir_{0.43}O_{3-\sigma}$**	6.57 m^2/g	370±2 mV	*Energy Environ. Sci.*, **2017**, 10, 2432

Supplementary references

1. Crumlin, E. J. *et al.* Surface strontium enrichment on highly active perovskites for oxygen electrocatalysis in solid oxide fuel cells. *Energy Environ. Sci.* **5**, 6081-6088 (2012).

2. Seitz, L. C. *et al.* A highly active and stable IrO_x /$SrIrO_3$ catalyst for the oxygen evolution reaction. *Science* **353**, 6303 (2016).

3. Feng, Z. X. *et al.* In situ studies of the temperature-dependent surface structure and chemistry of single-crystalline (001)-oriented $La_{0.8}Sr_{0.2}CoO_{3-\delta}$ perovskite thin films. *J. Phys. Chem. Lett.* **4,** 1512-1518 (2013).

4. Sing, K. S. The use of gas adsorption for the characterization of porous solids. *Colloids and Surfaces* **38**, 113-124 (1989).
